# Cellular and molecular landscape of mammalian sinoatrial node revealed by single-cell RNA sequencing

**DOI:** 10.1038/s41467-020-20448-x

**Published:** 2021-01-12

**Authors:** Dandan Liang, Jinfeng Xue, Li Geng, Liping Zhou, Bo Lv, Qiao Zeng, Ke Xiong, Huixing Zhou, Duanyang Xie, Fulei Zhang, Jie Liu, Yi Liu, Li Li, Jian Yang, Zhigang Xue, Yi-Han Chen

**Affiliations:** 1 Department of Cardiology, Shanghai East Hospital, Tongji University School of Medicine, Shanghai, 200120 China; 2 Key Laboratory of Arrhythmias of the Ministry of Education of China, Shanghai East Hospital, Tongji University School of Medicine, Shanghai, 200120 China; 3 Institute of Medical Genetics, Tongji University, Shanghai, 200092 China; 4 Department of Regenerative Medicine, Tongji University School of Medicine, Shanghai, 200092 China; 5 Translational Center of Stem Cell Research, Tongji Hospital, Tongji University School of Medicine, Shanghai, 200065 China; 6 Department of Pathology and Pathophysiology, Tongji University School of Medicine, Shanghai, 200092 China; 7 Reproductive Medicine Center, Tongji Hospital, Tongji University School of Medicine, Shanghai, 200065 China

**Keywords:** Transcriptomics, Cardiovascular biology

## Abstract

Bioelectrical impulses intrinsically generated within the sinoatrial node (SAN) trigger the contraction of the heart in mammals. Though discovered over a century ago, the molecular and cellular features of the SAN that underpin its critical function in the heart are uncharted territory. Here, we identify four distinct transcriptional clusters by single-cell RNA sequencing in the mouse SAN. Functional analysis of differentially expressed genes identifies a core cell cluster enriched in the electrogenic genes. The similar cellular features are also observed in the SAN from both rabbit and cynomolgus monkey. Notably, *Vsnl1*, a core cell cluster marker in mouse, is abundantly expressed in SAN, but is barely detectable in atrium or ventricle, suggesting that *Vsnl1* is a potential SAN marker. Importantly, deficiency of *Vsnl1* not only reduces the beating rate of human induced pluripotent stem cell - derived cardiomyocytes (hiPSC-CMs) but also the heart rate of mice. Furthermore, weighted gene co-expression network analysis (WGCNA) unveiled the core gene regulation network governing the function of the SAN in mice. Overall, these findings reveal the whole transcriptome profiling of the SAN at single-cell resolution, representing an advance toward understanding of both the biology and the pathology of SAN.

## Abstract (web summary)

The spontaneous bioelectrical activity of pacemaker cells in sinoatrial node (SAN) triggers the heartbeats. Here, the authors perform single-cell RNA sequencing in the mouse SAN and identify molecular and cellular features of the SAN conserved in rabbit and cynomolgus monkey, identifying a new potential SAN marker.

## Introduction

The sinoatrial node (SAN) is the pacemaker of the heart in mammals. The spontaneous bioelectrical activity of a relatively small population of pacemaker cells in SAN initiates the heart beat and controls heart rates^1–3^. The SAN is a small, specialized and heterogeneous myocardial structure located at the junction of the superior vena cava and right atrium^4^. The pacemaker activity of SAN cells originates from the synergistic effect of ion channels, transporters, and Ca^2+^ regulators^3,5,6^. In addition, it is tightly regulated by autonomic nerve signaling^7^.

Both histologically and functionally, SAN presents significant heterogeneity. In terms of cell morphology, cells in different regions of SAN show a variety of shapes. It has been observed clinically for a long time that there is a “leading pacemaker” site within the SAN, and the “leading pacemaker” site can drift around in SAN, suggesting that bioelectrical activity of SAN pacemaker cells is not uniform^8–10^. There is an urgent need to reveal systematically the cellular taxonomic basis and molecular regulatory networks that make up these heterogeneities.

Recently, Linscheid et al.^11^ systematically analyzed the proteomics of SAN biopsies in mice, and then linked the measured protein abundances to specific cell types by performing SAN single-nucleus RNA sequencing (snRNA-seq). However, the characteristic and regulatory network of SAN pacemaker cells, the key cells generating spontaneous rhythm, need further investigation. With the advantage of single-cell RNA sequencing (scRNA-seq) more expressed genes can be detected and used for comprehensive analysis of cell subtype and regulatory network at transcriptome level. Now we are more likely to identify cellular heterogeneity and explore differential gene expression patterns in a tiny and complex tissue like the SAN^12^. Properties of SAN in adult mammals, such as low cell number and large cell size, have restricted analysis of its whole transcriptomics characteristics at single-cell resolution by high-throughput methods. We obtained SAN single cells from different mammalian species by manual cell picking, then proceeded scRNA-seq to depict the gene expression pattern of SAN.

In the present study, we demonstrated the molecular panorama of SAN cell clusters and the core molecular regulation network underlying the SAN pacemaker activity. Our study classified mouse SAN cells into four clusters, including one core cell cluster, and similar cellular features were also observed in SAN from both rabbit and cynomolgus monkey. We identified a marker *Vsnl1*, which was highly and specifically expressed in the mouse SAN but rarely detected in the atrium or ventricle. Functional studies showed that *Vsnl1* played a pivotal role in regulating the frequency of electrical activity in mouse SAN. Moreover, the consistency of its abundant expression in SAN across species makes it a possible candidate marker gene in other two species as well.

## Results

### Distinct clusters of SAN cells in adult mouse

**Fig. 1:**
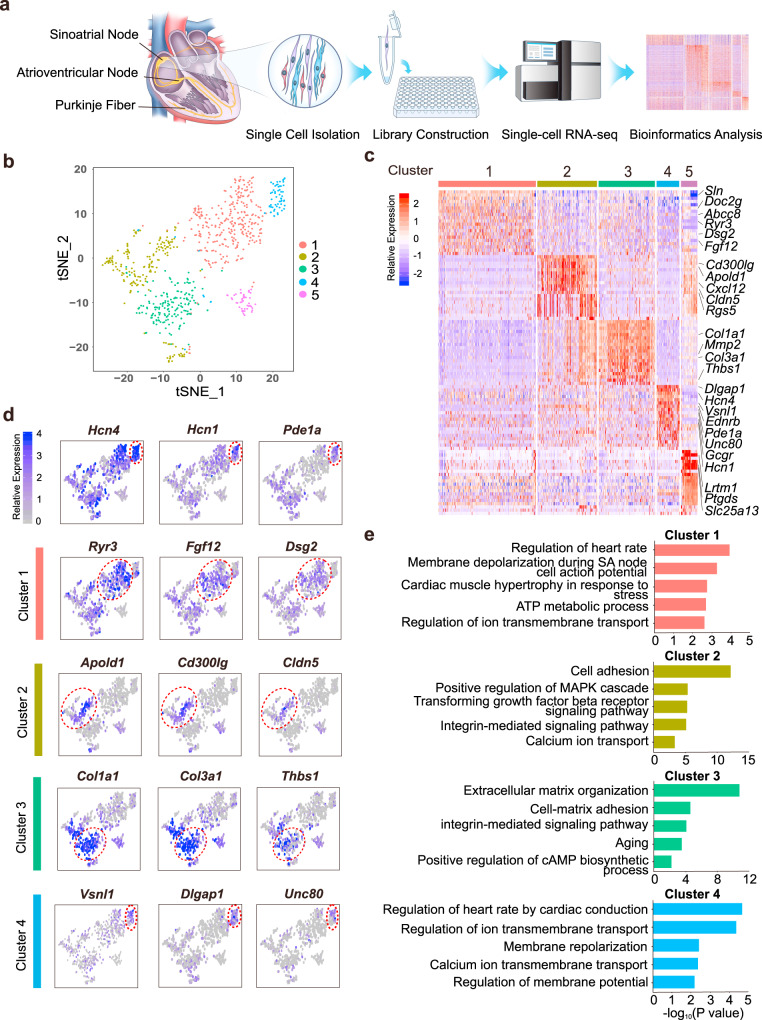
Classification and functional analysis of mouse sinoatrial node (SAN) cells. **a** Illustrator and workflow for single-cell RNA sequencing (scRNA-seq) of SAN. **b** *t*-distributed stochastic neighbor embedding (t-SNE) analysis identified five clusters including four SAN cell clusters (Clusters 1, 2, 3, 4) and one atrial and ventricular (AV) cardiomyocytes cell cluster (Cluster 5) (*n* = 771 biologically independent cells of 25 independent animals). **c** Heat map shows top differentially expressed genes (DEGs) of each cluster. Cluster 4 was highly expressed cell markers including *Hcn4, Hcn1*, and *Ednrb, Pde1a* and identified as the core cell cluster. **d** Feature plot of DEGs shows *Vsnl1, Unc80*, and *Dlgap1* were expressed more specifically than *Hcn4* and *Hcn1* in Cluster 4. Location of each cluster was marked with the red circle. **e** Gene Ontology (GO) terms show the biological processes of SAN cell clusters and Cluster 4 was mainly associated with regulation of heart rate and ion transmembrane transport, while Cluster 1, 2, and 3 were all related to electrical activity but had certain difference.

We isolated and pooled 771 cells, including 718 cells from microdissected SAN tissues of 21 adult mice and 53 atrial and ventricular (AV) cardiomyocytes from 4 adult mice, then performed scRNA-seq using the Smart-seq2 protocol^12,13^ (Fig. 1a and Supplementary Fig. 1) to examine their transcriptomes. We achieved an average of nine million reads per cell and an average of 7586 genes per cell was detected. The gene expression was quantified using trimmed clean reads. Clustering analysis of cells was determined based on the expression of 2500 mouse heart-related genes previously reported by Vedantham et al.^6^ (Supplementary Data 1). The *t*-distributed stochastic neighbor embedding (t-SNE) analysis revealed four distinct, previously undescribed cell clusters within the mouse SAN (Clusters 1–4) and one AV cell cluster (Cluster 5) (Fig. 1b and Supplementary Figs. 2 and 3). The distinct cell clusters of SAN were identified based on the functional analysis of differentially expressed genes (DEGs) of each cluster (Fig. 1c and Supplementary Data 2). Cluster 1 highly expressed genes involved in calcium ion transport, regulation of heart rate, and membrane depolarization of SAN cells. In addition to expressing pacemaker cell markers, Cluster 2 and Cluster 3 were enriched in genes for cell adhesion and extracellular matrix organization, respectively. Cluster 4 highly expressed classical markers of pacemaker cells and the basic electrogenic genes involved in SAN pacemaker activity including ion channels, transporters, Ca^2+^ regulators, and autonomic nerve signaling (Fig. 1d, e). Cluster 1 is similar to Cluster 4 but with relatively lower expression of classic markers and less rhythm related genes. We therefore speculated that Cluster 4 might be the core cell cluster and have more important roles in SAN function compared to other clusters.

DEG analysis showed that Cluster 4 highly expressed known markers for SAN including *Hcn4* and *Hcn1* (refs. ^14,15^). Besides ion channels, two genes related to autonomic nervous system regulation, *Ednrb* and *Pde1a*, were also highly expressed in this cluster (Fig. 1c, d). *Pde1a*, a cyclic nucleotide phosphodiesterase which regulates cAMP metabolism, drives SAN pacemaker function and indices of heart rate variability^16^. These cell markers indicated that this population might be the core cell cluster, representing the presumed pacemaker cells with the highest pacing frequency. In addition, Gene Ontology (GO) enrichment analysis of DEGs confirmed that Cluster 4 is associated with the regulation of heart rate (Fig. 1e). Notably, we detected three genes, namely *Vsnl1* (a member of the visinin/recoverin subfamily of neuronal calcium sensor proteins), *Unc80* (Unc-80 homolog, NALCN channel complex subunit), and *Dlgap1* (the disks large-associated protein 1), which were relatively highly and specifically expressed in Cluster 4 compared to other clusters (Fig. 1d), suggesting their utility as potential markers of SAN core cells. Cluster 1 highly expressed genes of calcium ion transport (*Ryr3, Doc2g, Dsg2, Fgf12, Abcc8*, and *Sln*); Cluster 2 expressed genes involved in cell adhesion and multiple cell signaling pathways (*Apold1, Cd300lg, Cxcl12, Cldn5*, and *Rgs5*); Cluster 3 highly expressed extracellular matrix and collagen markers related genes (*Col1a1, Col3a1, Mmp2*, and *Thbs1*) (Fig. 1c, d). GO analysis indicated that the DEGs enriched in each cluster corresponded to their characteristic biological processes (Fig. 1e).

**Fig. 2:**
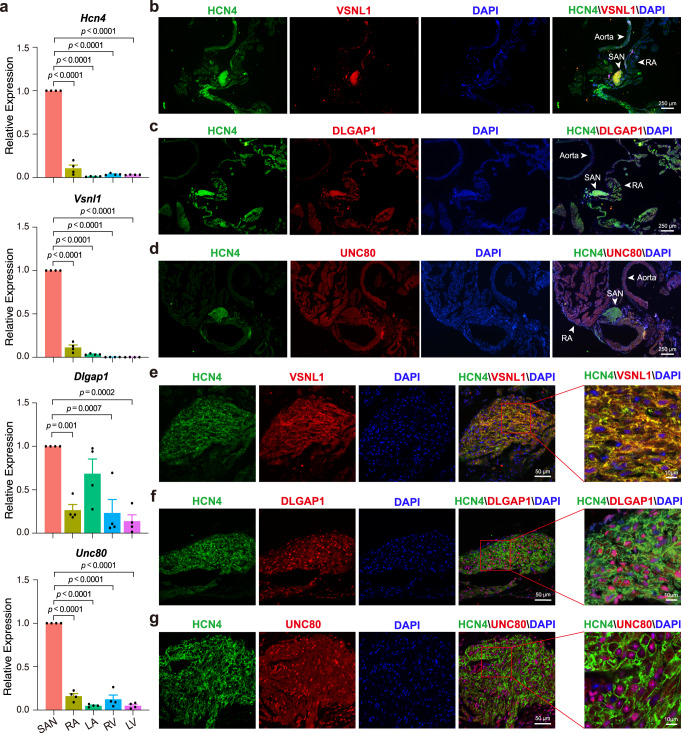
Expression pattern of the core cluster markers *Vsnl1*, *Dlgap1*, and *Unc80* in mouse heart. **a** qPCR analysis shows the expression of *Hcn4, Vsnl1, Dlgap1,* and *Unc80* in sinoatrial node (SAN), atrial, and ventricular tissue, respectively (*n* = 4 biologically independent animals per group), Dunnett’s multiple comparisons test, data are represented as mean ± s.e.m., adjusted *p* value was labeled on the top. RA right atrium, LA left atrium, RV right ventricle, LV left ventricle. **b**–**g** Immunohistochemical analysis of VSNL1, DLGAP1, and UNC80 in SAN slices at low magnification. Representative images are shown from *n* = 3 biologically independent samples. **b** Immunofluorescence images of HCN4 (green), VSNL1 (red), and DAPI (blue). **c** Immunofluorescence images of HCN4 (green), DLGAP1 (red), and DAPI (blue). **d** Immunofluorescence images of HCN4 (green), UNC80 (red), and DAPI (blue). **b**–**d** The arrows indicate the tissues surrounding the SAN. Scale bar = 250 μm. **e**–**g** Localization of the VSNL1, DLGAP1, and UNC80 within SAN at high magnification. **e** Immunofluorescence images of HCN4 (green), VSNL1 (red), and DAPI (blue). **f** Immunofluorescence images of HCN4 (green), DLGAP1 (red), and DAPI (blue). **g** Immunofluorescence images of HCN4 (green), UNC80 (red), and DAPI (blue). Scale bar = 50 μm. **e**–**g** Zoom images (the red box regions in the merged image) show the higher magnification. Scale bar = 10 μm.

**Fig. 3:**
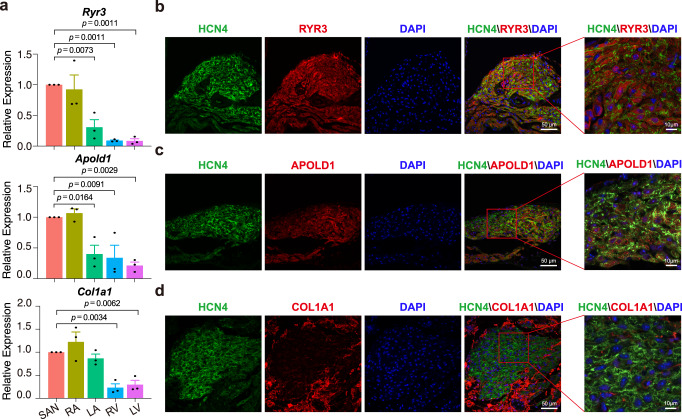
The expression pattern of non-core cluster genes *Ryr3, Apold1*, and *Col1a1* in mouse heart. **a** qPCR shows the expression of *Ryr3* (Cluster 1), *Apold1* (Cluster 2), and *Col1a1* (Cluster 3) in sinoatrial node (SAN), atrial, and ventricular tissue, respectively (*n* = 3 biologically independent animals per group). Dunnett’s multiple comparisons test, data are represented as mean ± s.e.m., adjusted *p* value was labeled on the top. RA right atrium, LA left atrium, RV right ventricle, LV, left ventricle. **b**–**d** Immunostaining shows the location of RYR3 (**b**), APOLD1 (**c**), and COL1A1 (**d**) in mouse SAN tissue, respectively. Representative images are shown from *n* = 3 biologically independent samples. Scale bar = 50 μm. Zoom images (the box regions in the merged images) show the higher magnification. Scale bar = 10 μm.

To investigate the expression pattern of these cell cluster marker genes in the heart, we dissected the mouse SAN, atrium, and ventricle tissues and performed qPCR (Figs. 2a and 3a). We found that the expression pattern of *Vsnl1*, *Dlgap1*, and *Unc80* was similar to that of *Hcn4*, which is relatively highly expressed in SAN compared to atrium and ventricle (Fig. 2a), though the transcriptional level of *Unc80* was lower than that of *Vsnl1* and *Dlgap1* in SAN (Supplementary Fig. 4). We then analyzed VSNL1, DLGAP1, and UNC80 protein expression in SAN by immunofluorescence. The results showed that though VSNL1 and DLGAP1 were ubiquitously distributed throughout the SAN, while they exhibited heterogeneous expression in different regions within SAN (Fig. 2b, c, e, f). Consistent with the mRNA level, the low protein expression of UNC80 was observed in SAN (Fig. 2d, g). Notably, VSNL1 was more specifically expressed than DLGAP1 and UNC80 in SAN, and was almost undetectable in atrium and ventricle (Fig. 2a–d and Supplementary Fig. 5). In addition, other three cell cluster markers, such as RYR3 (Cluster 1), APOLD1 (Cluster 2), and COL1A1 (Cluster 3), were all readily detected in SAN (Fig. 3b–d), demonstrating the reliability of our scRNA-Seq data.

### The SAN cell clusters can be segregated from the AV cell cluster

In addition to the mouse SAN cells, we simultaneously analyzed AV cells, which have no pacemaker activity and were well-segregated based on their transcriptomes into a distinct cluster (Cluster 5) (Fig. 1b and Supplementary Fig. 3a). Moreover, several genes, such as *Lrtm1*, *Ptgds,* and *Slc25a13*, were exclusively and highly expressed in Cluster 5, suggesting their utility as specific markers of AV cells to distinguish them from SAN cells (Fig. 1c and Supplementary Fig. 3b). We then profiled the expression of distinctive ion channels in Cluster 5 (AV cell cluster) and Cluster 4 (SAN core cell cluster). These ion channel genes were differentially expressed in atrioventricular and SAN tissues according to previous reports^11,17,18^. In general, the gene expression profiles distinguished these two cell clusters as expected. We found that Cluster 5 was characterized by higher expression of *Gja5* and *Gja1*, and lower expression of *Cacna1g*, *Cacna1g* and the auxiliary Ca^2+^ channel subunits *Cacna2d2* whereas Cluster 4 was characterized by higher expression of *Hcn1* and *Hcn4*, and lower expression of *Gja1*. However, we noted that the expression of *Cacna1c* and *Ryr2*, which was thought to be slightly lower in SAN tissues than in atria and ventricles^18^, was almost identical in Cluster 5 and Cluster 4. *Slc25a13*, which is associated with calcium ion binding, was highly and exclusively expressed in Cluster 5 compared to Cluster 4, so were *Lrtm1* and *Ptgds* (Supplementary Fig. 3c).

### Vsnl1 is involved in the regulation of SAN function

**Fig. 4:**
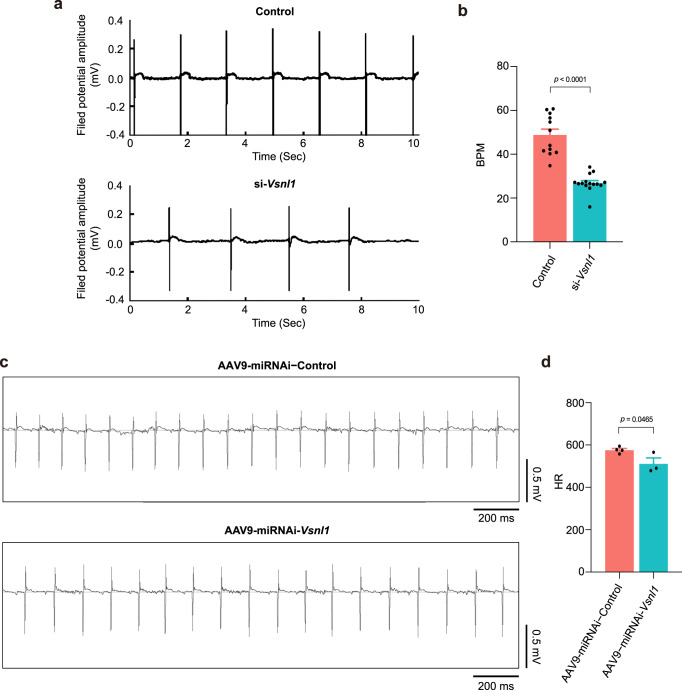
The core cell cluster marker *Vsnl1* governs the pacing activity. **a**, **b** *Vsnl1* deficiency reduced the beating rate of human induced pluripotent stem cell-derived cardiomyocytes (hiPSC-CMs). **a** Representative field potential traces in hiPSC-CMs transfected with nonsense siRNA (Control) or *Vsnl1* targeting (si-*Vsnl1*) siRNA. **b** Statistic data of beating rates. Control: *n* = 12 independent examinations over three independent experiments; si-*Vsnl1*: *n* = 15 independent examinations over three independent experiments. BPM beats per minute. **c**, **d** *Vsnl1* deficiency reduced the heart rate of mouse. **c** Representative electrocardiographs (ECGs) obtained from conscious mice through telemetric recording system. **d** Statistic data of mouse heart rate (HR). AAV9-miRNAi-Control: *n* = 4 biologically independent animals; AAV9-miRNAi-*Vsnl1*: *n* = 3 biologically independent animals. Unpaired, two-tailed Student’s *t*-test, data are represented as mean ± s.e.m., *p* value was labeled on the top (**b, d**).

To explore the possible role of distinct cell clusters in SAN, we interfered several marker genes in each cluster on human induced pluripotent stem cell-derived cardiomyocytes (hiPSC-CMs) to record changes in beating rate with microelectrode array (MEA). Our results showed that *Vsnl1* deficiency reduced the beating rate of hiPSC-CMs by 44.8% of the control (Fig. 4a, b), suggesting it is involved in the regulation of cellular autonomic rhythm. However, the knockdown of marker genes in other three clusters did not alter this parameter (Supplementary Fig. 6). To further verify the effects of *Vsnl1* on SAN function in vivo, we measured the heart rate in conscious mice after AAV-mediated gene knockdown with AAV9-miRNAi*-Vsnl1* or AAV9-miRNAi-*Control* by the telemetry. In agreement with the MEA data, *Vsnl1* deficiency also reduced the heart rate of the mouse (Fig. 4c, d), validating its role in SAN function.

Next, we analyzed the effects of *Vsnl1* on molecules involved in bioelectrical activity, such as ion channels and Ca^2+^ regulators expression in both *Vsnl1* knockdown neonatal rat cardiomyocytes and mouse SAN. For the *Vsnl1* knockdown neonatal rat cardiomyocytes, at transcription level, the expression of the voltage-dependent Ca^2+^ channels Ca_v_3.3 (*Cacna1i*) and Ca^2+^-transporters (*Serca2* and *Ryr2*) was downregulated, whereas there is no statistical difference in the expression of other channels, including voltage-dependent K^+^ channels (*Kcna4*, *Kcnh2*, *Kcnj3*, *Kcnj5,* and *Kcnj11*), HCN channels (*Hcn4*), voltage-gated Na^+^ channels Na_v_1.5 (*Scn5a*), voltage-dependent Ca^2+^ channels Ca_v_1.2 and Ca_v_1.3 (*Cacna1c* and *Cacna1d*), and Ca^2+^-regulators (*Ncx1*) (Supplementary Fig. 7). We specifically extracted SAN regions of AAV9-miRNAi*-Vsnl1* and AAV9-miRNAi-*Control* mouse to determine the interference efficiency of *Vsnl1* and analyze these gene expression. The expression of *Vsnl1* significantly reduced in the SAN of AAV9-miRNAi-*Vsnl1* mouse (Supplementary Fig. 8). In AAV9-miRNAi*-Vsnl1* SAN, besides *Hcn4*, the transcriptomic expression of *Cacna1d*, *Cacna1i*, and *Serca2a* were significantly downregulated, with no statistical changes in the expression of *Kcna4*, *Kcnh2*, *Kcnj3*, *Kcnj5*, *Kcnj11*, *Scn5a*, *Cacna1c*, *Ryr2*, and *Ncx1* (Supplementary Fig. 8). The knockdown of VSNL1 and HCN4 has also been shown in AAV9-miRNA-*Vsnl1* SAN section with VSNL1 and HCN4 antibody co-staining (Supplementary Fig. 9), consistent with the decreased mRNA expression of *Vsnl1* and *Hcn4* in AAV9-miRNA-*Vsnl1* SAN (Supplementary Fig. 8).

### Gene expression network of SAN cells in adult mouse

**Fig. 5:**
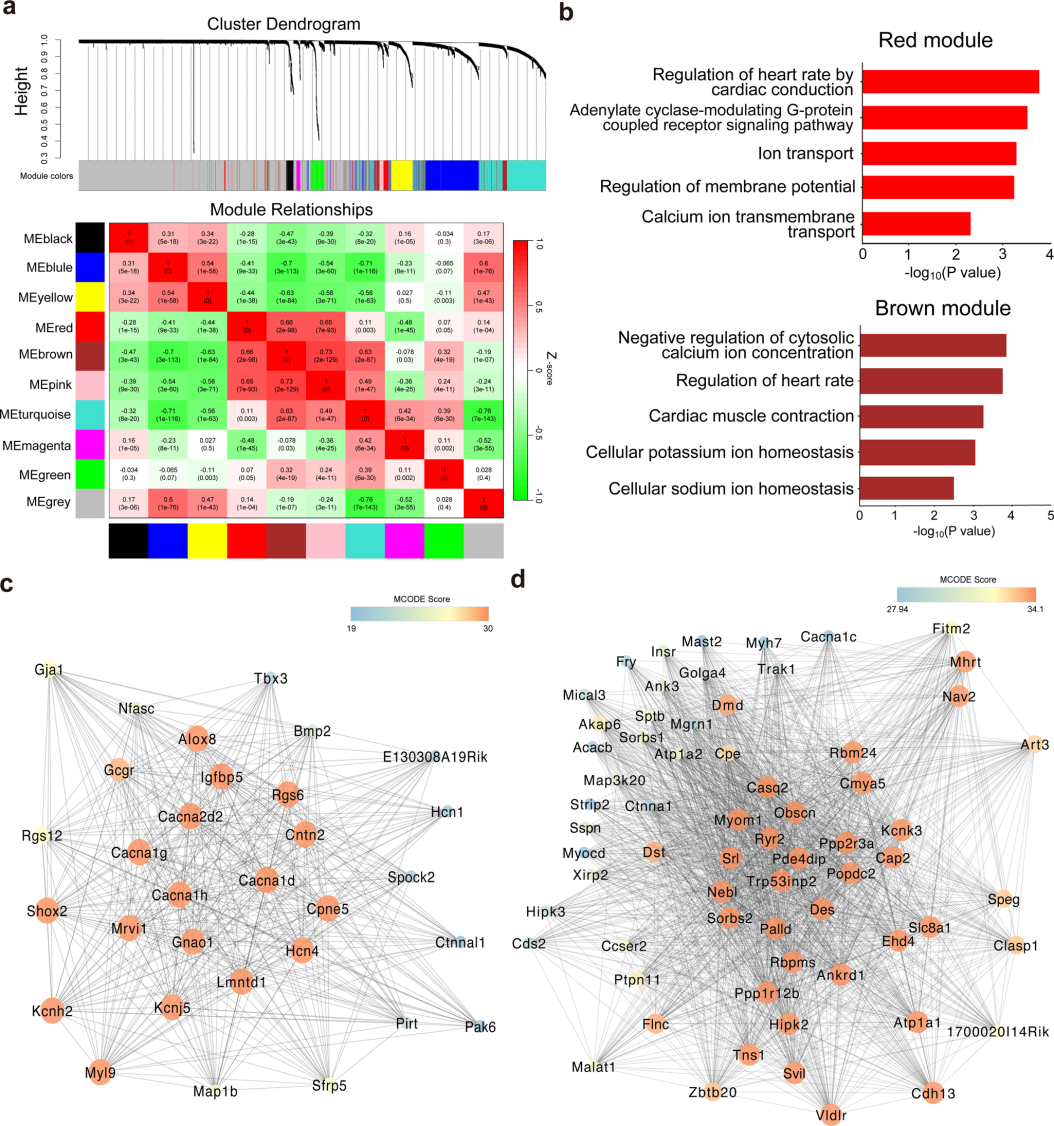
Weighted gene co-expression network analysis (WGCNA) analysis of mouse sinoatrial node (SAN) cells. **a** The hierarchical cluster dendrogram identifies ten gene co-expression modules. Heat map shows the correlation of these modules. **b** Gene Ontology (GO) analysis shows that the main terms of red and brown modules were all related to SAN pacemaker activity. **c** Co-expression network of red module genes mainly focus on K^+^ and Ca^2+^ channels. **d** Co-expression network of brown module genes mainly focus on Ca^2+^ regulators. The networks were created by Cytoscape and the high MCODE score of genes was mapped to the bright color of nodes.

To investigate gene expression programs governing SAN pacemaker activity, weighted gene co-expression network analysis (WGCNA) was performed to identify distinct modules of gene expression in the scRNA-seq data (Fig. 5a). We identified ten co-expression modules, six out of which were related to the regulation of ion channels. Notably, we found that two modules (red and brown) were mainly enriched in GO terms of heart rate, cardiac conduction, membrane potential, and ion transport (Fig. 5b). These two modules were comprised of three different classes of key genes associated with heart rhythm (Supplementary Data 3). The first class is ion channel genes, such as *Hcn4 and Cacna1d. Hcn4* encodes the hyperpolarization-activated, cyclic nucleotide-gated channel responsible for the hyperpolarization-activated or funny current (*I*_f_), which has a key role in the pacemaker potential of SAN^18,19^. *Cacna1d* encodes the voltage-gated L-type Ca^2+^ channel Ca_v_1.3, which activates during the diastolic depolarization phase of SAN pacemaker cells and may constitute the predominant voltage-dependent mechanism contributing to pacemaking^2,20^. Loss-of-function of Ca_v_1.3 in both mouse and humans causes sick sinus syndrome characterized by severe bradycardia^21–23^. The second class is transporter genes, such as *Slc8a1* and *Atp1a1. Slc8a1* encodes Na^+^/Ca^2+^ exchange 1 (NCX1), which is a major actor in cardiac cell Ca^2+^ homeostasis and is responsible for Ca^2+^ efflux during action potential repolarization^2,24^. Intact NCX1 activity is necessary for maintaining automaticity of pacemaker cells^25,26^. *Atp1a1* encodes Na^+^/K^+^ pump (Na^+^/K^+^-ATPase), which acts as a transporter to maintain intracellular ion homeostasis and generates a net outward current that influences cellular pacemaking^27,28^. The third class is Ca^2+^ regulator genes, such as *Ryr2* and *Camk2a. Ryr2* encodes the intracellular Ca^2+^ channels ryanodine receptor 2 (RYR2), activated by Ca^2+^ influx due to subsarcolemmal local Ca^2+^-induced Ca^2+^ release (LCICR), which controls chronotropic state of SAN pacemaker cells and is the predominant mechanism of SAN automaticity “Ca^2+^ clock” hypothesis^2^. *Camk2a* encoding Ca^2+^/calmodulin-dependent protein kinase II (CaMKII) is a multi-functional serine/threonine kinase targeting a large number of substrates, including ion channels, pumps, Ca^2+^ cycling proteins, and transcription factors. In SAN, CaMKII controls pacemaker cell activity by regulating the activation and reactivation kinetics of L-type Ca^2+^ channels, thus affecting pacemaker cell activity^2,29^. Moreover, autonomic nerve signaling/G protein-coupled receptor genes (*Gnao1, Rxfp1*, and *Gcgr*) and transcription factor genes (*Shox2*, *Tbx3* and *Bmp2*) were identified in these two modules as well. In general, the co-expression network of the red module mainly contains K^+^ channels and Ca^2+^ channels genes (Fig. 5c), while the brown module tends to contain more Ca^2+^ regulator genes (Fig. 5d).

Predicated protein–protein interaction of the red and brown modules reflected the similar biological processes with GO analysis, and the construction of interaction network showed the connectivity of the ion channels, transporters, Ca^2+^ regulators, autonomic nerve signaling/G protein-coupled receptors, and transcription factors in these two critical modules (Supplementary Fig. 10a, b). Interestingly, we found that the core cell cluster gene *Gcgr* (encoding the glucagon receptor, GCGR), which was involved in GPCR signaling and cAMP biosynthetic process through interaction with *Rxfp1* and *Adcy5* (Fig. 1c and Supplementary Fig. 10a, c), was also identified as a hub gene in the co-expression network of the red module (Fig. 5c). Other related modules, including the blue, yellow, pink, and turquoise modules, possessed a high correlation coefficient and represented genes involved in cell adhesion, extracellular matrix organization, and cell signaling pathways (Supplementary Fig. 10d).

### Distinct clusters and gene expression network of SAN cells in rabbits

**Fig. 6:**
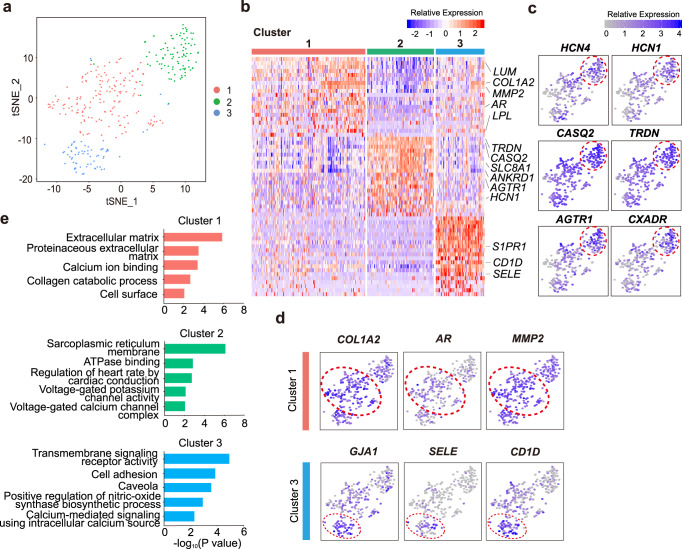
Cell classification of rabbit sinoatrial node (SAN) cells. **a** t-distributed stochastic neighbor embedding (t-SNE) analysis identifies three-cell clusters in rabbit SAN (*n* = 343 biologically independent cells of six independent animals). **b** Heat map shows differentially expressed genes (DEGs) of each cluster. **c** Feature plot of specific DEGs in Cluster 2. **d** Feature plot of specific DEGs in Cluster 1 and Cluster 3. Location of different clusters was marked with the red circles. **e** Gene Ontology (GO) analysis of three clusters. Cluster 2 showed it is highly associated with regulation of heart rate, while Cluster 1 was enriched in extracellular matrix and Cluster 3 was related to cell adhesion besides ion channels regulation.

We next set to compare the SAN gene expression patterns between mouse and rabbit. We isolated 343 SAN cells from six adult rabbits and performed scRNA-seq as described above to detect cell clusters of gene expression. The distinct cell clusters of rabbit SAN were identified mainly using the same criteria as described in mouse. Among three clusters found in rabbit SAN cells (Fig. 6a and Supplementary Fig. 11), Cluster 2 highly expressed ion channel and transporter genes (*HCN4, HCN1, ATP1A1*, and *SLC8A1*), Ca^2+^ regulator genes (*ATP2A2, RYR2, CXADR, CASQ2, TRDN*, and *ANKRD1*), and the angiotensin II receptor (*AGTR1*) which are of functional relevance to SAN pacemaker activity, thus cluster 2 was defined as the core cluster in rabbit SAN. Meanwhile, Cluster 1 highly expressed genes of the androgen receptor (*AR*), Ca^2+^-binding proteins (*LPL* and *LUM*), extracellular matrix (*COL1A2* and *MMP2*), and ion channels. Cluster 3 highly expressed genes of cardiac gap junction genes (*KCNJ8*, *KCNE4*, and *GJA1*), autonomic nerve signaling/G protein-coupled receptor genes (*S1PR1* and *EDNRB*), and cell adhesion genes (*SELE* and *CD1D*), respectively (Fig. 6b–d, Supplementary Fig. 11 and Supplementary Data 4). GO enrichment analysis demonstrated that Cluster 2 was mainly related to regulation of heart rate by cardiac conduction; meanwhile, the biological function of Clusters 1 and 3 were also confirmed by DEG analysis in each cluster (Fig. 6e). Interestingly, *CXADR*, encoding the coxsackie and adenovirus receptor which is predominantly expressed at the intercalated discs of cardiomyocytes^30,31^, was highly expressed in Cluster 2 (Fig. 6c). Though the role of *CXADR* in SAN has not been demonstrated, we speculate that it may be involved in the conduction between SAN cells. We further investigated the transcriptional level of the core cell cluster markers in different regions of rabbit heart, the results showed that these genes (*HCN4*, *HCN1*, *CASQ2*, *TRDN*, *AGTR1*, *CXADR*) were expressed relatively specific in the SAN (Supplementary Fig. 12). Notably, *VSNL1*, the core cell cluster marker in mouse, was also highly and specifically expressed in the rabbit SAN (Supplementary Fig. 13).

WGCNA analysis identified three modules of gene co-expression in rabbit SAN (Supplementary Fig. 14a). The turquoise module contains key genes encoding ion channels, transporters, Ca^2+^ regulators, autonomic nerve signaling/G protein-coupled receptors and transcription factors, including *HCN4, HCN1*, *KCNH2*, *CACNA1C*, *CACNB2*, *ATP1A1*, *ATP1B1*, *SLC8A1, ATP2A2, RYR2, PLN, CASQ2, TRDN, AGTR1, EDNRA, BMP2*, and *FOXP2* (Supplementary Data 5). Transporters and Ca^2+^ regulator genes constructed a co-expression network (Supplementary Fig. 14b). Protein interaction analysis verified the key role of the turquoise module in SAN (Supplementary Fig. 14c).

### Distinct clusters of SAN cells in adult cynomolgus monkey

**Fig. 7:**
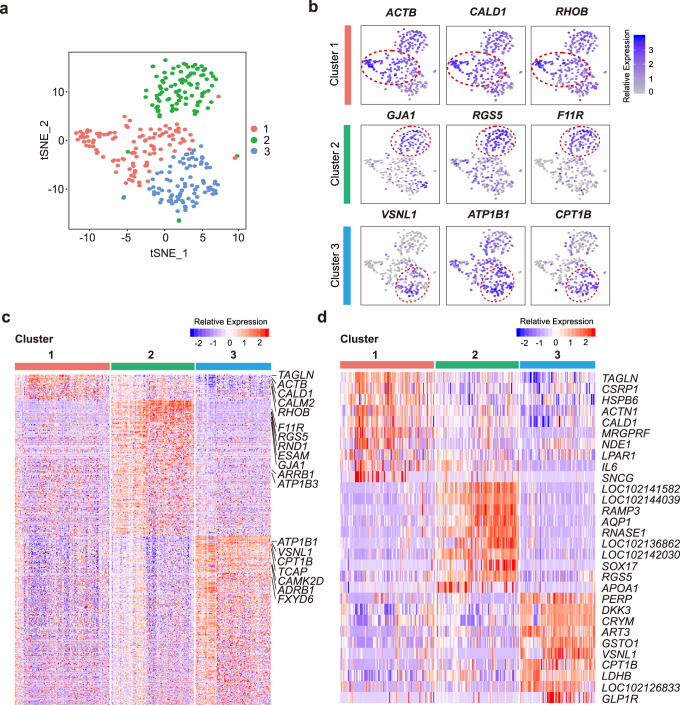
Clustering analysis of cynomolgus monkey sinoatrial node (SAN) cells. **a** Three clusters of monkey SAN cells were identified by *t*-distributed stochastic neighbor embedding (t-SNE) (*n* = 296 biologically independent cells of 12 independent animals). **b** Feature plot of specific differentially expressed genes (DEGs) in each cluster and Cluster 3 was identified as the core cell cluster. *VSNL1* was also specifically expressed in Cluster 3. Location of different clusters was marked with the red circle. **c** Heat map shows all DEGs of each cluster. The main DEGs of each cluster were indicated in the right column. **d** Heat map shows top10 specific DEGs of each cluster.

Then we isolated 296 SAN cells from 12 adult cynomolgus monkey and performed transcriptional clustering analysis as described in mouse and rabbit. The SAN cells of the cynomolgus monkeys were segregated into three clusters (Fig. 7a), and functional analysis of DEGs indicated that Cluster 3 was the core cell cluster based on high expression of ion channel-related genes (*ATP1B1, VSNL1, CAMK2D, TCAP,* and *FXYD6*) and autonomic nervous system regulation genes (*ADRB1* and *CPT1B*). *VSNL1*, which was highly and specifically expressed in mouse and rabbit SAN, was also the TOP DEGs in Cluster 3, further verified that this cluster is the core cell cluster in monkey. Besides Cluster 3, Cluster 1 was defined by high expression of genes of the Ca^2+^ regulator (*CALD1 and CALM2*), G protein-coupled receptor signaling molecular (*RHOB*) and cytoskeletal protein (*TAGLN* and *ACTB*), and ion channel, Cluster 2 highly expressed cardiac gap junction genes (*ATB1B3* and *GJA1*), autonomic nerve signaling/G protein-coupled receptor genes (*ARRB1, RND1* and *RGS5*), and cell adhesion genes (*F11R* and *ESAM*) (Fig. 7b–d and Supplementary Data 6). It should be noted that numerous functional genes related to SAN function were not enriched in the DEGs of primate SAN clusters compared with mouse and rabbit, which may be due to the limited annotation of cynomolgus monkey genome. Many SAN-related genes were merely predicted, thus were not included in clustering analysis.

### The key pacemaker activity genes expression analysis across species

**Fig. 8:**
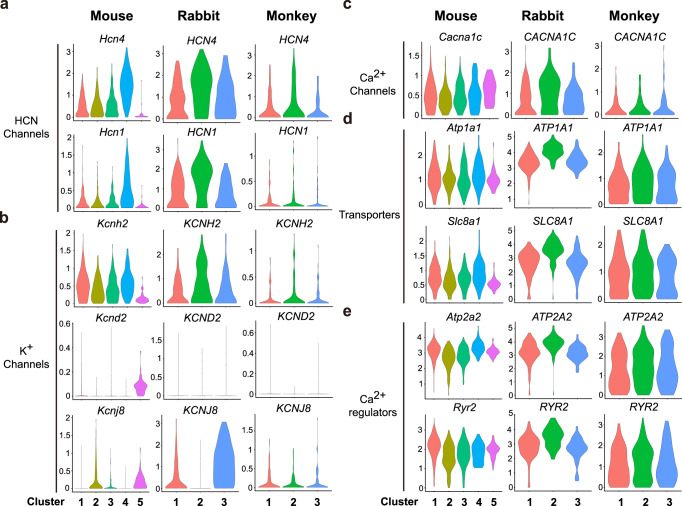
Relative expression of key functional gene related to autonomic rhythm of sinoatrial node (SAN) cells among three species. **a** Expression of HCN channel genes. **b** Expression of K^+^ channel genes. *Kcnd2* was detected in atrial and ventricular (AV) cells but low expression in SAN cells. **c** Expression of Ca^2+^ channel genes. **d**, **e** Expression of transporter and Ca^2+^ regulator genes.

Finally, to determine the species specificity of key gene expression patterns in different mammals, we further compared the expression of several functional genes associated with pacemaker activity common to mice, rabbits and cynomolgus monkey. We found that genes encoding HCN channels, K^+^ channels, Ca^2+^ channels, transporters and Ca^2+^ regulators are all expressed in SAN of the three species assayed (Fig. 8). In contrast, the expression of *Kcnd2* (encoding a potassium voltage-gated channel Kv4.2) was low in SAN cells of three species (Fig. 8b). Interestingly, *Kcnj8*, encoding the inwardly rectifying K_ATP_ channel subunit Kir6.1, which has been reported to be present in mouse SAN pacemaker cells and involved in the regulation of heart rate^32^, was not expressed in the potential core cell cluster of mouse and rabbit SAN (Fig. 8b).

## Discussion

The field of SAN research has long been dominated by two major questions. First, are there different subsets of pacemaker cells in SAN? Second, what is the core molecular regulatory network of pacemaker activity in SAN? In this study, we preliminarily answered these two questions by transcriptionally profiling the SAN cells using scRNA-seq. On the one hand, we found that the SAN cells in mice can be divided into four clusters, including one core cell cluster, whereas SAN cells in rabbit and cynomolgus monkey can be divided into three clusters, each of which also contains a core cell cluster. On the other hand, we identified the core gene regulation network of pacemaker activity, which was consistent with the protein interaction analysis. The genes regulatory network underlying the pacemaker activity may govern the spontaneous depolarization and intrinsic automaticity of SAN pacemaker cells.

One of the bottlenecks in SAN pacemaker activity research is lack of specific cell-type markers. *Hcn4* and *Hcn1*, the classical molecular markers of SAN^33,34^, are highly expressed in the SAN and cardiac pacemaker regions of different species and provides great convenience for the study of pacemaker cell biology and electrophysiology. The DEGs analysis in our study revealed three genes, including *Vsnl1*, *Dlgap1*, and *Unc80*, could be the possible candidate markers of core cell cluster in mouse SAN. However, the expression of *Dlgap1* and *Unc80* was less specific and relatively low abundance in SAN. We found that *Vsnl1* was highly and specifically expressed in SAN compared to atrium and ventricle, functionally, it participates in cardiac cell rhythm regulation. Moreover, *VSNL1* was also highly and specifically expressed in the rabbit SAN, and was one of the TOP DEGs in the core cell cluster of the monkey, suggesting the conservation of its abundant expression in SAN across species. All these make *Vsnl1* a possible SAN marker. In addition, markers of AV cells not only define AV cells themselves but play an important complementary role in distinguishing SAN cells from AV cells. Our study showed that *Lrtm1*, *Ptgds*, and *Slc25a13* were highly expressed in AV cells and rarely expressed in the SAN cells. When combining the AV specific genes with SAN specific expression of *Vsnl1*, it will be benefit to the identification of the SAN cells. Furthermore, the specific nature of the markers identified here may lead to lineage tracing for the development of SAN cells.

The *Vsnl1* gene encodes visinin-like 1 protein, which belongs to the visinin-like protein subfamily of neuronal calcium sensor proteins. VSNL1 has been found to be involved in calcium signaling pathways and implicated in synaptic pathology in Alzheimer’s disease^35^. A recent study analyzed the transcriptomes of late fetal FACS-purified Tbx3^+^ SAN nodal cells and Nppb-Katushka^+^ AV chamber cardiomyocytes, and identified a series of SAN-enriched gene program including *Vsnl1*, which was also found enriched in fetal human SAN regions^36^, suggesting its potential role in SAN development. However, its function in SAN remains unclear. Our study revealed the effect of *Vsnl1* in SAN through functional studies and gene expression analysis. We speculated that the Vsnl1^+^ core cells may have more important roles in SAN function compared to other clusters. Moreover, considering that the Vsnl1^+^ core cells are potentially diffusely distributed in SAN tissue, they may be the cellular basis of the “leading pacemaker” site and “pacemaker shift” phenomenon based on their non-restricted distribution within SAN. The specific expression of *Vsnl1* in the SAN of different species may shed light on its research in human. The role of *Vsnl1* in SAN function needs to be fully established in the future study. Our findings also open avenues in the investigation of lineage tracing of pacemaker cells, cardiac autonomic rhythm and biological pacemaker.

The hiPSC-CM model was used to investigate the effect of cell cluster marker genes on frequency in vitro. However, the hiPSC-CM is primarily used as an approach to study cellular phenotypes of inheritable cardiac diseases and provide in vitro platforms for mechanistic investigation in regenerative medicine^37–39^. In the field of cardiac electrophysiology, the hiPSC-CM could be used as a cellular model for some pacemaker mechanism studies because there are some similarities between hiPSC-CMs and pacemaker cells such as the presence of spontaneous firing rate and the reactivity to isoproterenol and nifedipine^40,41^. However, hiPSC-CMs cannot be justified as surrogate for SAN pacemaker cells. hiPSC-CMs compose mixed cardiac cell types showing immature phenotypes, small action potentials, and fetal-like transcriptome profiles; importantly, they also exhibit heterogeneous electrophysiology phenotypes and spontaneous automaticity^42^. Furthermore, the regulation mechanisms underlying the pacing of SAN cells and hiPSC-CMs are different as well. The pacing of SAN cells is thought to be initiated and maintained by a membrane clock and a Ca^2+^ clock, whereas the initiation and frequency of spontaneous pacing in hiPSC-CMs is thought to be mainly depends on localized spontaneous Ca^2+^ releases via RYR and other Ca^2+^ regulators such as SERCA2 and NCX, which could be independent of membrane depolarizations generated by ion channels^43–45^.

The gene expression analysis after *Vsnl1* knockdown suggests the different mechanisms of *Vsnl1* in frequency regulation in different cell types. The pluripotent stem cells derived SAN pacemaker-like cell would be a more ideal model for exploring the ionic basis of SAN function in vitro. However, most cardiac differentiation models based on pluripotent stem cells are used to simulate ventricular cardiomyocytes, whereas approaches for the generation of SAN-like pacemaker cells are sparse with low efficiency^39,46^. The development of sophisticated differentiation protocol to efficiently produce pacemaker-like cells will definitely bring an opportunity to the study of SAN.

The intrinsic rhythm of SAN is regulated by the autonomic nervous system^47,48^ as well, although in ways still poorly understood to date. In this study, autonomic nerve signaling/G protein-coupled receptor genes were identified as important elements of pacemaker activity in core gene regulatory network. Notably, *KCNJ5*, encoding an inwardly rectifying potassium channel Kir3.4, was included in the core gene regulatory network. Kir3.4 is responsible for the acetylcholine/adenosine-induced potassium *I*_KACh_^49^. The vagal release of acetylcholine stimulates muscarinergic activation of an inhibitory G protein, which in turn opens the Kir3.4 channel, the SAN pacemaker cells are then repolarized or hyperpolarized by activating *I*_*KACh*_^50^. Interestingly, a specific glucagon receptor *G*cgr, which has been linked to the regulation of heart rate^51,52^, was also present in core gene regulatory network and may be involved in the regulation of G protein-coupled receptor signaling in SAN, suggesting the non-ion channel genes may critically affect SAN function as well. The results of WGCNA could also provide directional and systematic strategy for mechanistic investigation.

One of the exciting aspects of current SAN research is the relationship between transcription factors and SAN development^53^. In this study, it was found that two transcription factor genes, i.e., *Shox2* and *Tbx3*, which are essential for the formation and function of cardiac conduction system^53^, are members of the core gene regulatory network of adult SAN cells. These transcription factors interact with and co-regulate each other. For example, *Shox2* expression is crucial for SAN development and is restricted to the sinus venosus, where it prevents the formation of working myocardium by inhibiting *Nkx2-5* expression and activating *Hcn4*, *Isl1*, and *Tbx3* expression^54,55^. Our WGCNA data revealed that these transcription factors and the relevant regulatory network may also play an important role in SAN pacemaker activity.

The basic proteins involved in SAN pacemaker activity mainly include ion channels, transporters, Ca^2+^ regulators, autonomic nerve signaling/G protein-coupled receptors, and transcription factors^3,10,55^. Our work shows that these types of components all exist to some extent in the core gene regulation network governing the function of SAN cells in different species. Therefore, the classical theoretical framework of pacemaker cell autonomic rhythm may have potential species universality. However, it must be pointed out that, in terms of specific hub regulatory molecules, there is a degree of difference among the three species analyzed in this study. For instance, in rabbit, high expression of transporter genes and Ca^2+^ regulator genes are more obvious in core cell clusters (Fig. 8d, e). Thus, caution should be exercised in analyzing the mechanisms underlying SAN pacemaker activity across species.

In summary, our work reveals the molecular and cellular maps of SAN cells at single-cell resolution, providing systematic data for the origin and regulation of SAN pacemaker activity research. These findings are of fundamental significance in the in-depth understanding of biology and pathogenesis of SAN, as well as its interventions^56^.

## Methods

### Animals

This study conformed to the rules of the Guide for the Care and Use of Laboratory Animals made by the U.S. National Institutes of Health. All of the animal experiments were approved by the Animal Care and Use Committee of Tongji University School of Medicine (mouse and rabbit experiments), or Institutional Animal Care and Use Committee of Suzhou Xishan Zhongke Drugs Research and Development Centre (monkey experiments). Breedings of mice were kept under SPF conditions in a 12 h dark–light-cycle environment with food and water ad libitum. Ambient temperature was 22 °C and humidity 60%.

### Isolation of single cells

Single SAN cells were isolated from male adult mice, New Zealand White rabbits, and cynomolgus monkeys, respectively, based on previously described methods^13,57,58^. The heart was removed quickly and placed in the pre-warmed Tyrode’s solution containing: 140 mM NaCl, 5.4 mM KCl, 1.2 mM KH_2_PO_4_, 1.8 mM CaCl_2_, 1.0 mM MgCl_2_, 5.5 mM glucose, and 5 mM HEPES (pH was adjusted to 7.4 with NaOH). The solution was continuously oxygenated with O_2._ The SAN was dissected from the region bordered by the crista terminalis, the superior and inferior vena cavae. The SAN tissue was then cut into small pieces and washed twice in low-Ca^2+^ solution containing: 140 mM NaCl, 5.4 mM KCl, 1.2 mM KH_2_PO_4_, 0.2 mM CaCl_2_, 18.5 mM glucose, 50 mM taurine, and 1.0% BSA (pH was adjusted to 6.9 with NaOH). The SAN was then digested at 36–37 °C for 20–25 min in the low-Ca^2+^ solution containing elastase (0.3 mg/ml; Worthington, NJ, USA), collagenase type 2 (0.8 mg/ml; Worthington, NJ, USA), and protease (0.13 mg/ml; Sigma, Chemical Co.) with gentle agitation. The concentration of the enzyme was adjusted to achieve an optimized digestion in different species. The digestion was stopped by transferring the pieces to the modified Kraftbruhe (KB) solution, containing 100 mM potassium glutamate, 10 mM potassium aspartate, 25 mM KCl, 10 mM KH_2_PO_4_, 2 mM MgSO_4_, 20 mM taurine, 5 mM creatine, 0.5 mM EGTA, 20 mM glucose, 5 mM HEPES, and 1.0% BSA (pH was adjusted to 7.2 with KOH). The SAN pieces were then dissociated by pipetting gently in the KB solution with a fire-polished glass pipette.

The AV myocytes were isolated as previously described^59^. The heart was removed and perfused with a low-Ca^2+^ solution containing 117 mM NaCl, 4 mM KCl, 10 mM HEPES, 1 mM KH_2_PO_4_, 4 mM NaHCO_3_, 1.7 mM MgCl_2_, 0.1 mM CaCl_2_, and 10 mM glucose. Then, the heart was perfused with low-Ca^2+^ solution containing 300 U/ml collagenase type 2 (Worthington, NJ, USA) for 15–20 min. When the heart became slightly pale and flaccid, the atrium and ventricle were removed and cut into small pieces in the KB solution, respectively. The tissue was dissociated through pipetting and then filtered through a cell strainer (100 μm; BD Falcon, NJ, USA).

### Single-cell RNA sequencing

Single-cell RNA sequencing was performed using Smart-seq2 protocol with minor modification^60,61^. In brief, single cell was pipetted into 0.5 μl lysis buffer under the microscope to release the total RNA. RNA was reversed to first-strand cDNA through nine cycles using Superscript III (Invitrogen, 18080044) and then was amplificated for 18 PCR cycles using KAPA polymerase (Kapa Biosystems, KK2601). The RT-PCR product was purified using Agencourt AMPure XP Beads (Beckman Coulter, A63881), and then 0.1 ng cDNA was used for Nextera tagmentation (Illumina, FC-131-1096) to construct library. The ERCC spike-in (Invitrogen, 4456740) was added in reverse transcription mix during the library construction according to the Smart-seq2 protocol. Finally, the libraries were sequenced with paired end 2 × 150 bp reads on Illumina HiSeq X10.

### Single-cell RNA sequencing data processing

Raw sequencing reads from single-cell RNA sequencing libraries were trimmed to remove adapter sequences, which removed the leading and trailing low-quality bases below quality 3 or N bases, cut the sliding window which average quality per base drops below 15, and dropped reads below the 36 bases long (Version Trimmomatic-0.33). The clean reads were aligned to the mouse genome (GRCm38 assembled by UCSC), the rabbit genome (oryCun2 assembled by UCSC), or cynomolgus monkey genome (macFas5 assembled by UCSC) with Hisat2 (version 2.0.5). The mapping rate was 32.32% when measured using the raw reads to align to mouse reference genome sequence, while the mapping rate is 42.35% when mapped to the mouse reference genome using the trimmed reads. The gene annotation file for mouse was downloaded from the database GENCODE (release 20)^62^. Gene annotation files of cynomolgus monkey and rabbit obtained from UCSC were organized into the RefSeq Genes tracks using the table tool, respectively. Unique mapped reads were counted with HTSeq (version 0.9.1)^63^ and grouped according to the sample name. For a given cell, the number of reads was counted up within a gene. This read count quantitation was used to estimate the gene expression of each gene in the cells and the read count matrix was used for the subsequent analysis. Single cells with more than 200 genes detected were used for downstream analysis.

The key functional genes described in Fig. 8 are predicted genes, which are not annotated in RefSeq Genes tracks in cynomolgus monkeys. We extracted these genes using ENSEMBL database and aligned them to macFas5 to calculate the read count. For mouse, the relative expression of functional genes was calculated in the clusters separately.

### Identification of cell clusters and DEGs

Raw counts were used as the input of Seurat R package (version 2.3). In the process, count data were normalized and log-transformed by “NormalizeData” function, which normalized the read counts for each cell by the total gene expression through multiplies this value by a scale factor (10,000) and log-transforms the result. Then the normalized read counts were used in the downstream analysis. Overall, using “FindVariableGenes” function, 7733 highly variable genes were obtained to perform principal component analysis (PCA). After that, significant PCs were identified through the “JackStraw” function with default parameters. Fourteen PCs were selected as the significant components for t-SNE analysis. We then set the clustering parameter resolution to 0.6 for the “FindClusters” function in Seurat to identify cell clusters. Among the clusters, DEGs were identified using the function “FindMarkers” and “FindAllMarkers” of Seurat Package with min.pct set to 0.25. Top DEGs were presented according to the log fold-change of the average expression (avg.logFC) and adjusted *p* value (p.val.adj.). Finally, GO enrichment analysis was performed using DAVID (version 6.8).

For mouse, we screened out 2500 genes which are enriched in mouse heart basing on previously report and performed the cell cluster analysis^6^. The expression data of these genes were extracted and processed as described above.

### Weighted gene co-expression analysis

The read count data were pre-processed before WGCNA analysis (version 1.64). First, the read counts with less than 10 were filtered out to remove low-expressed features and then 3831 genes left. Then, the read counts of these filtered 3831 genes were normalized with the function “Variance Stablizing Transformation” (VST) in DESeq2 package (version 1.22.2).

The normalized data were clustered to examine possible obvious outliers. Then we use the “pickSoftThreshold” function to choose a proper soft-thresholding power for network construction. The power 4 was found to be the lowest power for which the scale-free topology fit index curve flattens out upon reaching a high value. Based on this power, a signed weighted correlation network was constructed and modules were identified. The parameter mergeCutHeight (0.25) was used for merging of modules.

Module eigengenes were used to measure the module membership (also known as module eigengene based connectivity kME). Genes with highest module membership values were referred as intramodular hub genes (kME > 0.8). Intramodular hub genes were centrally located inside the module and represented the expression profiles of the entire module.

Cytoscape (version 3.6.1) was used to visualize the gene connections in different modules. Using the MCODE (version 1.5.1) to find the network of a cluster, which contained 31 nodes and 417 edges in whole mouse red module genes (Score: 27.8), and this network was laid out using “Prefuse Force Directed Layout” according to the weight. The visualization of the network was set to the high MCODE score of gene nodes with the large size and the bright color. The mouse brown module and rabbit turquoise module were analyzed using the same way. The cluster (Score: 48.123) contained 66 nodes and 1564 edges was found in the mouse brown module. The cluster in rabbit turquoise module (Score: 52.933) contained 61 nodes and 1588 edges.

STRING (version 11.0) was used for prediction and integrating the protein–protein interaction coding by module genes. Genes were inputted into the “Multiple Proteins” term and meaning of network edges was set to confidence. Hub proteins with its connected nodes were displayed.

### Quantitative real-time PCR

Total RNA of adult mouse SAN and atrium tissues was isolated using GenElute Single Cell RNA Purification Kit (Sigma, RNB300), total RNA of adult mouse ventricle, adult rabbit heart tissues, and neonatal rat cardiomyocytes were extracted using Trizol reagent (Thermo Fisher Scientific). Purified RNA was reverse-transcribed by the PrimeScript RT Reagent kit (Takara Bio, RR037A). Quantitative real-time PCR (qPCR) was performed using SYBR Green kit (TOYOBO CO, QPK-201) on the QuantStudio^TM^ 6 Flex system (Applied Biosystems; Thermo Fisher Scientific). GAPDH was used for endogenous control and the relative expression of mRNA level was quantified using 2^−^^△△CT^ method. The primers sequences are listed in Supplementary Data 7.

### Histology and immunofluorescence histochemistry

After anesthesia, the hearts of adult mice (8–12-week-old mice) were quickly removed, then atrium and peripheral connections were preserved. After rinsing with cold PBS, the hearts were fixed with 4% paraformaldehyde (PFA; Sigma) overnight at 4 °C. Next, hearts were dehydrated in increasing concentrations of ethanol and embedded in paraffin. Hearts were sectioned in 6 μm in horizontal section. The SAN of adult mice is located in the posterior wall of the right atrium, in the intercaval region adjacent to the atrial muscle of the crista terminalis, extending from the superior to near the inferior vena cava (Supplementary Fig. 1a). The SAN is surrounded by connective tissue, and the SAN node artery passing through the area. SAN area was labeled by staining HCN4, atrium was labeled by Connexin 43, HCN4 was abundantly present throughout the entire SAN area, while Connexin 43 was detected exclusively in the atrium (Supplementary Fig. 1b).

After deparaffinization, re-hydration, and microwaving antigen retrieval in citrate solution, paraffin-embedded heart slices were blocked with 10% goat serum (Invitrogen) and incubated with primary antibody overnight at 4 °C. Next day, the slices were washed in PBST for three times and then incubated with respective fluorescent secondary antibody (Invitrogen) for 1 h at room temperature. The slices were washed again in PBST for three times then stained with DAPI (Sigma) to label the nuclei. Primary antibodies included HCN4 (Sigma, SAB5200035, 1:50), Connexin 43 (CST, 3512, 1:50), VSNL1 (Gene Tex, GTX115039, 1:50), Collagen I (Abcam, ab21286, 1:50), DLGAP1 (Affbiotech, AF0308, 1:50), UNC80 (BIOSS, BS-12121R, 1:50), APOLD1 (Novus Biologicals, NBP2-58460, 1:50), RYR3 (Novus Biologicals, NBP2-76962, 1:50), Connexin 40 (Invitrogen, 37-8900, 1:50), cTNT (Abcam, ab8295, 1:50). Secondary antibodies included Goat anti-Mouse IgG Alexa Fluor® 488 (Abcam, ab150113, 1:200), Goat anti-Rabbit IgG Alexa Fluor® 555 (Abcam, ab150078, 1:200), and Goat anti-Mouse IgG Alexa Fluor® 633 (Sigma, SAB4600139, 1:200). Pictures were taken from a confocal microscope (Leica TCS SP8).

### Culture and transfection of hiPSC-derived CMs

hiPSC-derived CMs were purchased from Help Stem Cell Innovations (Nanjing, China). hiPSC- derived CMs were routinely maintained in cardiomyocytes culture medium (Help Stem Cell Innovations, HELP4001) and medium was changed every 48 h. After 18 days, hiPSC-derived CMs were dissociated with cardiomyocytes dissociation medium (Help Stem Cell Innovations, HELP4005) for 5 min at 37 °C, and then seeded onto MEA plates (5 × 10^5^ cell/plate). Cultures were maintained at 37 °C with 5% CO_2_. Transfection of small interfering RNA (siRNAs) was done with the lipofectamine RNAiMAX (Invitrogen, 13778-150), according to the manufacturer’s guidelines. All siRNA sequences are listed in Supplementary Table 1.

### MEA recordings

MEA assay was performed as previously described^41,64^. The hiPSC-derived CMs were seeded on a fibronectin-coated Maestro MEA 24-well plate at a density of 50,000 cells per well to obtain spontaneously beating monolayers. The hiPSC- derived CMs were then transfected with siRNA-targeting candidate genes (sequence provided in Supplementary Table 1) with RNAiMAX (Invitrogen, 13778-150). The electrical activity was recorded with Maestro system (Axion Biosystems Inc) at day 4 after transfection following a 15-min equilibration. The raw data were analyzed with Axion’s platform software to measure beat period (s). Statistical analysis was performed with GraphPad.

### Isolation and culture of neonatal rat cardiomyocytes

The neonatal rat cardiomyocytes were isolated from 1- to 3-day-old Sprague-Dawley rats. The ventricles were separated from hearts and cut into pieces (~1–3 mm^2^). Then these pieces were dissociated in calcium-free HBSS (Gibco, 14175095) containing 0.125 mg/ml trypsin (Gibco, 15090046), 0.1 mg/ml collagenase type IV (Sigma, C4-22-1G), and 10 mg/ml DNase II (Sigma, D8764). Digestion was performed at 37 °C and heart sections were continually stirred. The supernatant was then collected into HBSS containing 10% FBS every 5 min. The digestion procedure was repeated approximately 8–10 times until the tissues were completely digested. The supernatant was centrifuged at 1000 r.p.m. for 10 min and resuspended in DMEM (Gibco, 11965092) supplemented with 10% FBS and 100 mM 5-bromo-20-deoxyuridine (Sigma, B5002). The resuspended cells were passed through a cell strainer (100 μm, BD Falcon), and then seeded onto 100-mm plastic dishes for 2 h at 37 °C to remove fibroblasts. The isolated neonatal rat cardiomyocytes were plated on 1% gelatin-coated plastic culture dishes at an appropriate density. After 24 h, the medium was changed to low-serum medium, and the cells were cultured for 48 h then for use. The gene expression analysis after *Vsnl1* knockdown was performed in neonatal rat cardiomyocytes transfected with siRNA (*Vsnl1*) using Lipofectamine RNAiMAX (Invitrogen, 13778-150).

### AAVs generation

The *Vsnl1*-targeting miRs were designed using Invitrogen’s online software (Invitrogen’s RNAi Designer: http://rnaidesigner.thermofisher.com/rnaiexpress/design.do; Target Design Options: miR RNAi). According to the software, six miRNAi sequences targeting the different positions of *Vsnl1* gene (*Vsnl1*-miRNAi) and negative control sequence (*Control*-miRNAi) were designed, synthesized, and cloned into the AAV2/9-CMV_bGI-eGFP-miRNAi plasmid vectors. The pAAV2-CMV_bGI-eGFP-miRNAi (*Vsnl1*) and pAAV2-CMV_bGI-eGFP-miRNAi (*Control*) plasmids were constructed using the BLOCK-iT Pol II miR RNAi Expression Vector Kits (Invitrogen, K493500) and the microRNA-based silencing technique (Taitool Bioscience)^65^. Then the vectors were co-transfected with the VSNL1-overexpressed plasmid into HEK293 cells, respectively, and the interference efficiency of each sequence is detected by western blot. Of the six miRNAi sequences, two validated miRNAi sequences (Target sequence 1: TGAGTTCAATGAGCATGAGCT; Target sequence 2: AGTGGAGATGCTGGAGATTAT) targeting the different positions of *Vsnl1* gene were packaged into the virus vectors, respectively. The virus was then injected in adult male mice through tail vein, and their effectiveness was further confirmed in mouse SAN by qRT-PCR. The most effective virus (Target sequence 1: TGAGTTCAATGAGCATGAGCT) was chosen and the corresponding miRNAi sequence as follows: 5′-TGCTGAGCTCATGCTCATTGAACTCAGTTTTGGCCACTGACTGACTGAGTTCAGAGCATGAGCT-3′.

### The telemetric recordings

For virus-injected mice, following vectors were used: AAV2/9-CMV_bGI-eGFP-miRNAi (AAV9-miRNAi-*Vsnl1*) (titer: 1.92 × 10^13^ v.g./ml; Taitool Bioscience) and AAV2/9-CMV_bGI-eGFP-miRNAi (AAV9-miRNAi-*control*) (titer: 1.70 × 10^13^ v.g./ml; Taitool Bioscience). All viral vectors were aliquotted and stored at −80 °C until use. Adult male mice were injected with a dose of 2 × 10^11^ viral genome particles through tail vein. Fluorescence microscopy confirmed the virus infection in mouse SAN (Supplementary Fig. 15) and the *Vsnl1* knockdown was confirmed in SAN regions (Supplementary Figs. 8 and 9). The mice were implanted with telemetric transmitters (ETA-F10; Data Sciences International) under 1–2% isoflurane anesthetization 1 week after virus injection. Telemetric transmitters were implanted into the peritoneal cavity of the mice and the paired wire electrodes were placed over the thorax, as previously described^66,67^. Then the mice were housed in the single cage and exposed in 12 h dark/light cycle environment. Mice were given 2 weeks to recover from surgery before ECG recording. Recording and analysis parameters were set according to the manufacturer’s instructions using Ponemah software (Datasci International).

### Western blot

Adult rabbit SAN and AV tissue proteins were extracted by RIPA lysis buffer (Beyotime Biotechnology, P0013C) containing protease inhibitor cocktail (Roche, 04693132001). Equal total proteins (80 μg) were separated by 10% SDS-PAGE (Thermo Fisher Scientific, NP0315BOX), and then transferred onto PVDF membranes (Millipore, IPVH00010) The membranes were blocked with 5% nonfat milk in TBST buffer for 1 h at room temperature and then incubated with VSNL1 antibody (Gene Tex, GTX115039, 1:1000) and GAPDH (Proteintech, 60004-1-Ig, 1:8000) overnight at 4 °C. On the next day, the membranes were incubated with conjugated secondary antibody (Invitrogen, A32730; Invitrogen, A32735) for 1 h at room temperature and the bands were visualized using the Odyssey imager.

### Statistical analysis

Statistical analysis was performed with GraphPad Prism 8.0. The qPCR data were tested using Dunnett’s multiple comparisons test and unpaired, two-tailed Student’s *t*-test. The data are represented as the mean ± s.e.m.

### Reporting summary

Further information on research design is available in the Nature Research Reporting Summary linked to this article.

## Supplementary information

Supplementary Information

Description of Additional Supplementary Files

Supplementary Data 1

Supplementary Data 2

Supplementary Data 3

Supplementary Data 4

Supplementary Data 5

Supplementary Data 6

Supplementary Data 7

Reporting Summary

## Data Availability

The authors declare that the data supporting the findings of this study are available within the article and Supplementary Information files. The single-cell RNA-sequencing data have been deposited in the NCBI Sequence Read Archive (accession number of BioProject is PRJNA531288). All remaining data will be available from the corresponding author upon reasonable request. Source data are provided with this paper.

## Source data

Source Data

## Code availability

The codes used in this study are available in Github [https://github.com/xue-lab/scRNA-seq-of-sinoatrial-node].
