# Supplementary material for: Cellular and molecular landscape of mammalian sinoatrial node revealed by single-cell RNA sequencing
Source: Nat Commun. 2021 Jan 12;12:287. doi: 10.1038/s41467-020-20448-x (PMC7804277; doi:10.1038/s41467-020-20448-x)
Supplement: Supplementary file 1 — Supplementary Information [file 41467_2020_20448_MOESM1_ESM.pdf]

SUPPLEMENTARY INFORMATION

Cellular and molecular landscape of mammalian sinoatrial node revealed by single-cell RNA sequencing

Table of contents:

Supplementary Fig. 1

Supplementary Fig. 2

Supplementary Fig. 3

Supplementary Fig. 4

Supplementary Fig. 5

Supplementary Fig. 6

Supplementary Fig. 7

Supplementary Fig. 8

Supplementary Fig. 9

Supplementary Fig. 10

Supplementary Fig. 11

Supplementary Fig. 12

Supplementary Fig. 13

Supplementary Fig. 14

Supplementary Fig. 15

Supplementary Table 1

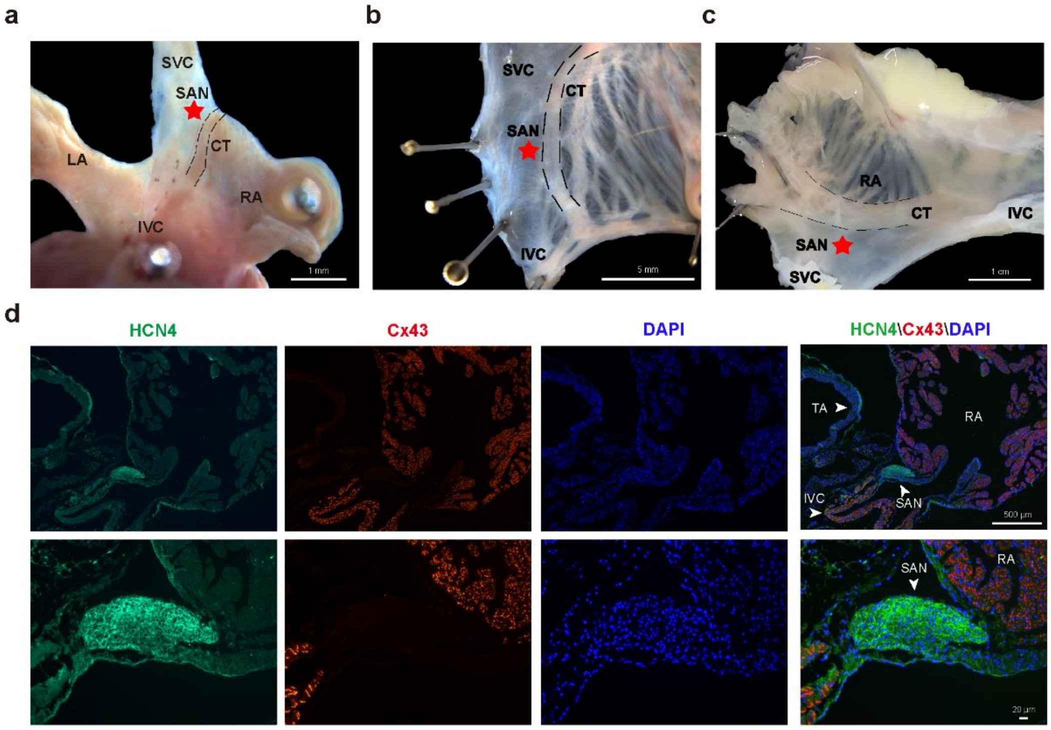

Supplementary Fig. 1. Illustration of sinoatrial node (SAN). **a-c** The SAN anatomy of different species: mouse (**a**), rabbit (**b**) and monkey (**c**). The red star indicates the SAN location. **a** scale bar = 1 mm. **b** scale bar = 5 mm. **c** scale bar = 1 cm. **d** Immunofluorescence labeling of mouse SAN. HCN4 green, Cx43 red, DAPI blue. Representative images are shown from n = 3 biologically independent samples. Top, scale bar = 500 µm. Bottom, scale bar = 20 µm. The arrows indicate the SAN and surrounding tissues. SVC, superior vena cava; IVC, inferior vena cava; CT, crista terminalis; LA, left atrium; RA, right atrium; TA, aorta.

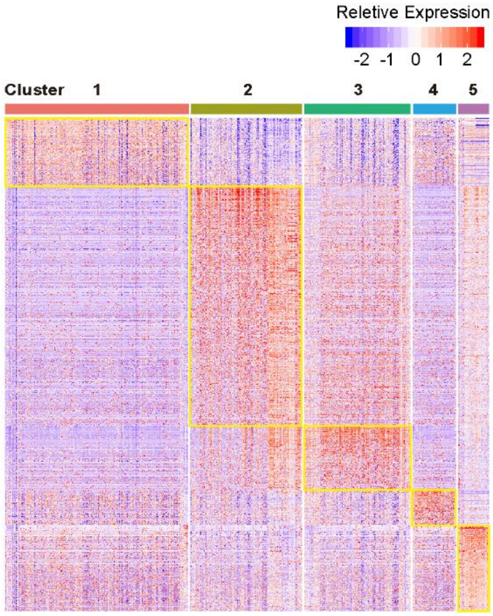

Supplementary Fig. 2. Heat map shows all differentially expressed genes (DEGs) of each cluster in mouse sinoatrial node (SAN) cells.

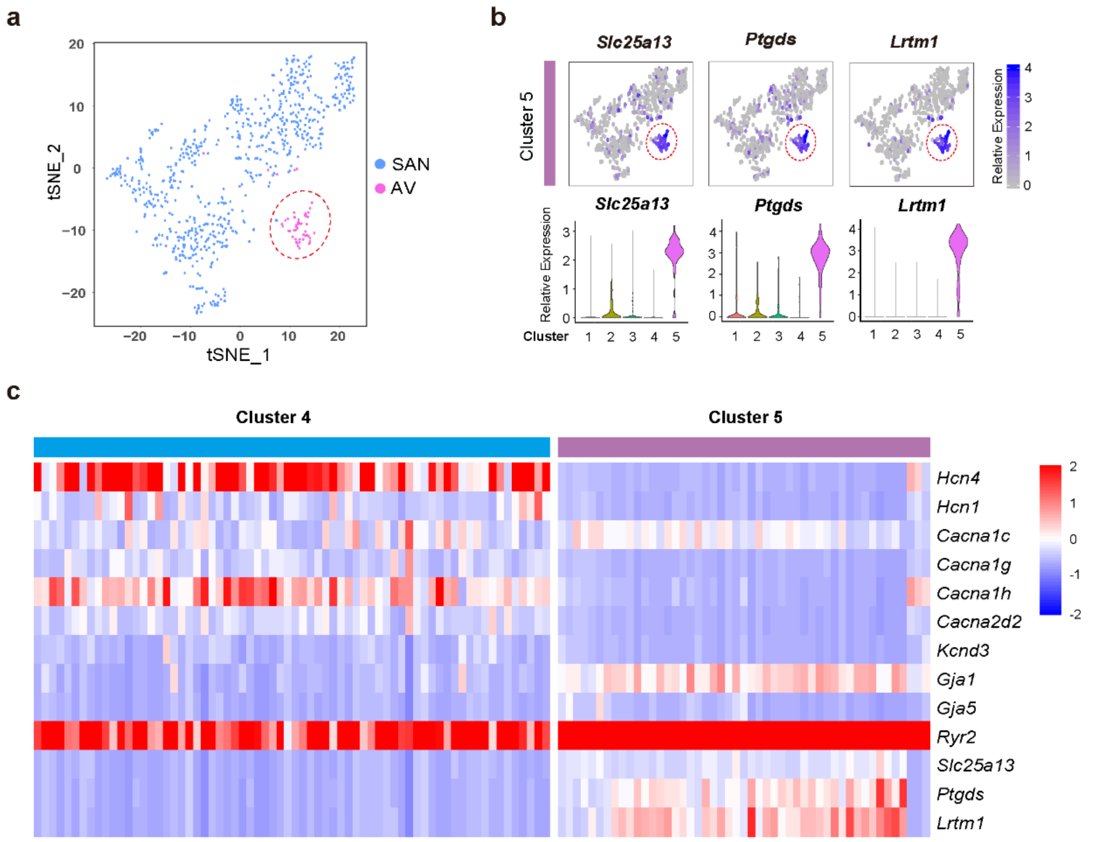

Supplementary Fig. 3. Expression of specific differentially expressed genes (DEGs) in atrial and ventricular (AV) cell cluster. **a** t-distributed stochastic neighbor embedding (tSNE) plot shows AV cells were well segregated with SAN cells and enriched in Cluster 5 (indicated by the red circle). **b** *Ptgds*, *Lrtm1* and *Slc25a13* were specifically expressed in AV cells (Cluster 5, indicated by the red circle), and they may be served as the markers for AV cells to distinguish from SAN cells. **c** Heatmap shows the expression of distinctive ion channels in Cluster 5 (AV cell cluster) and Cluster 4 (SAN core cell cluster).

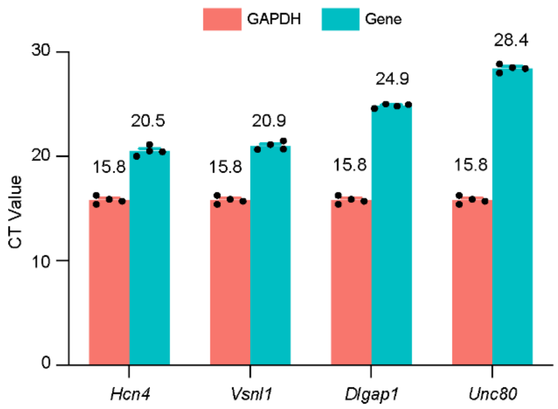

Supplementary Fig. 4. The average cycle threshold (CT) values of the core cell cluster markers *Hcn4*, *Vsnl1*, *Dlgap1* and *Unc80* in mouse sinoatrial node (SAN) (n = 4 independent animals per gene. Data are represented as mean ± sem.). *GAPDH* served as the reference gene. Average CT value of 28.4 suggested the relatively low-abundance transcription of *Unc80* in SAN compared to *Vsnl1* and *Dlgap1*.

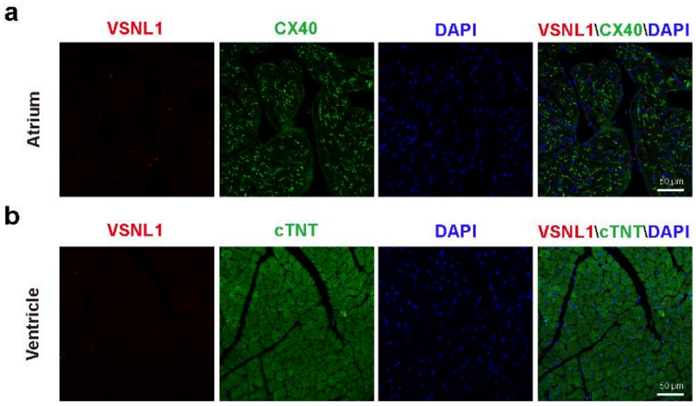

Supplementary Fig. 5. The fluorescence staining showed the low expression of VSNL1 in mouse atrium (**a**) and ventricle (**b**) tissues. Representative images are shown from n = 3 biologically independent samples. Red, VSNL1; green, CX40/cTNT; blue, DAPI. Scale bar =50 µm.

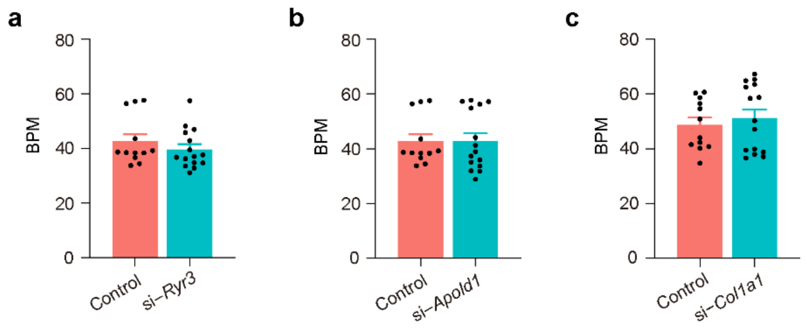

Supplementary Fig. 6. The deficiency of cluster marker genes *Ryr3* (**a**), *Apold1* (**b**) and *Col1a1* (**c**) had no effect on the beating rate (Beats per minute, BPM) in human induced pluripotent stem cell - derived cardiomyocytes (hiPSC-CMs). The *si-Ryr3* group and *si-Apold1* group were in the same batch with the common control group. n = 3 independent experiments. Unpaired, two tailed Student's t-test. Data are represented as mean ± sem.

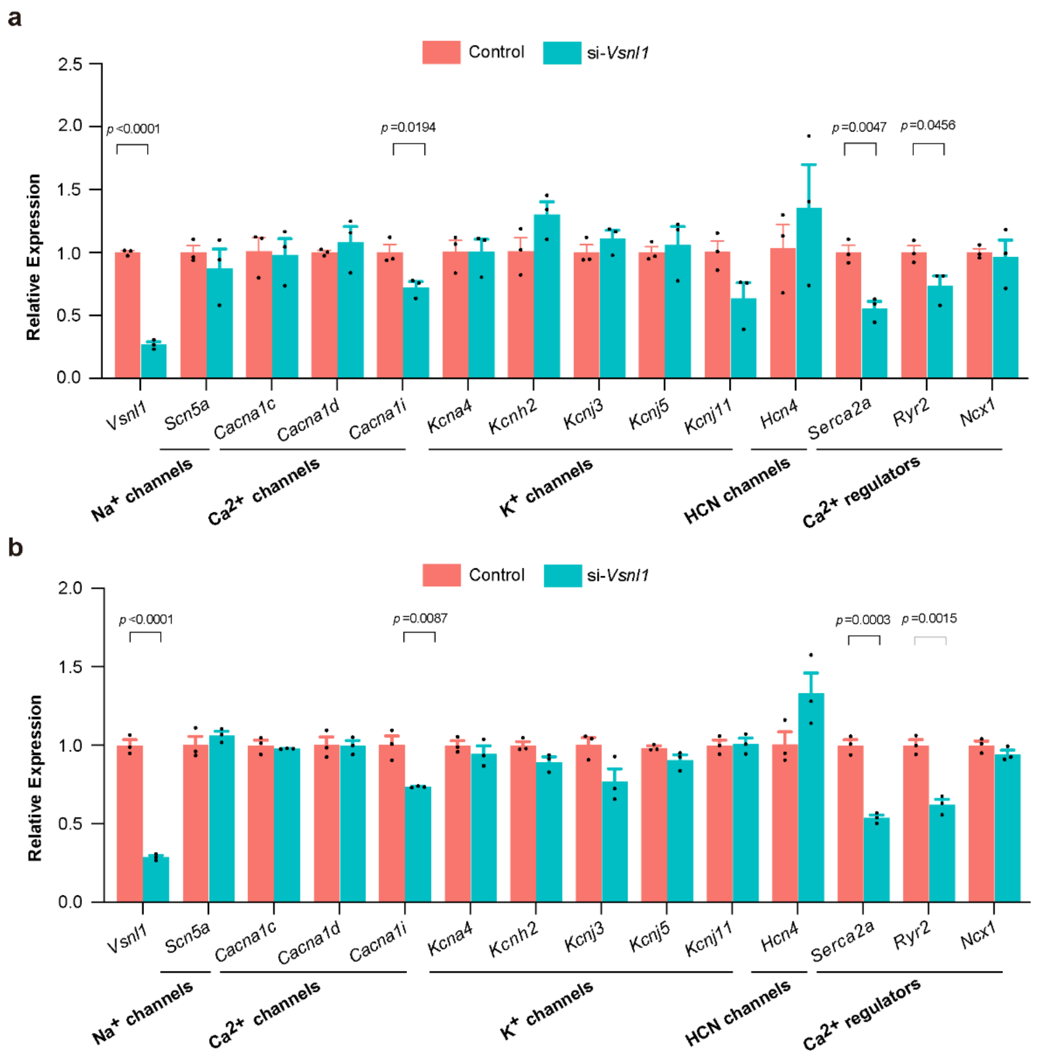

Supplementary Fig. 7. The transcriptome expression profile of Na^{+}, Ca^{2+}, K^{+}, HCN channels and Ca^{2+} regulator genes in *Vsnl1* knockdown neonatal rat cardiomyocytes using *Vsnl1*-1 (**a**) or *Vsnl1*-2 (**b**) siRNA sequences. n = 3 independent experiments. Unpaired, two tailed Student's t-test. Data are represented as mean ± sem.

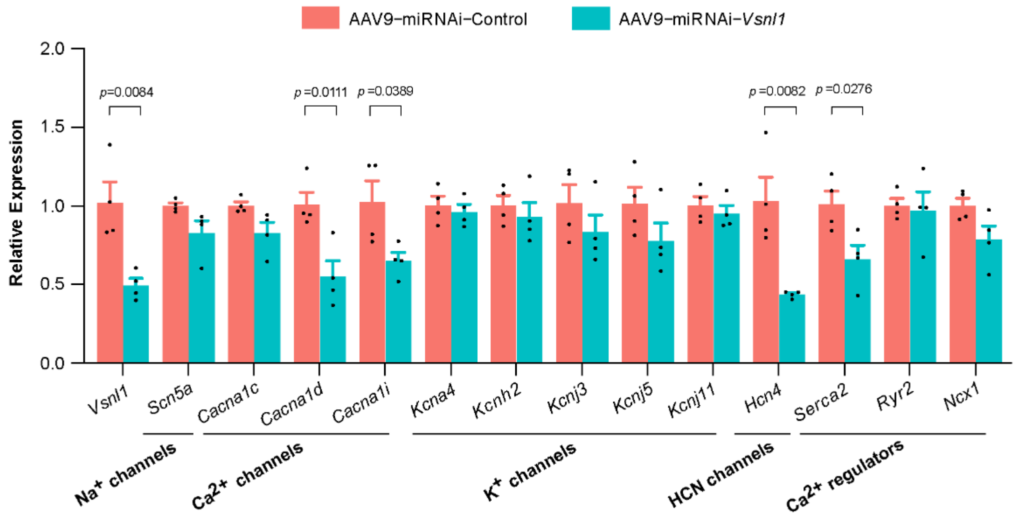

Supplementary Fig. 8. The transcriptome expression profile of Na^{+}, Ca^{2+}, K^{+}, HCN channels and Ca^{2+} regulator genes in sinoatrial node (SAN) of AAV9-miRNA-*Vsnl1* mouse and AAV9-miRNAi-Control mouse. n = 4 biologically independent animals. Unpaired, two tailed Student's t-test. Data are represented as mean ± sem.

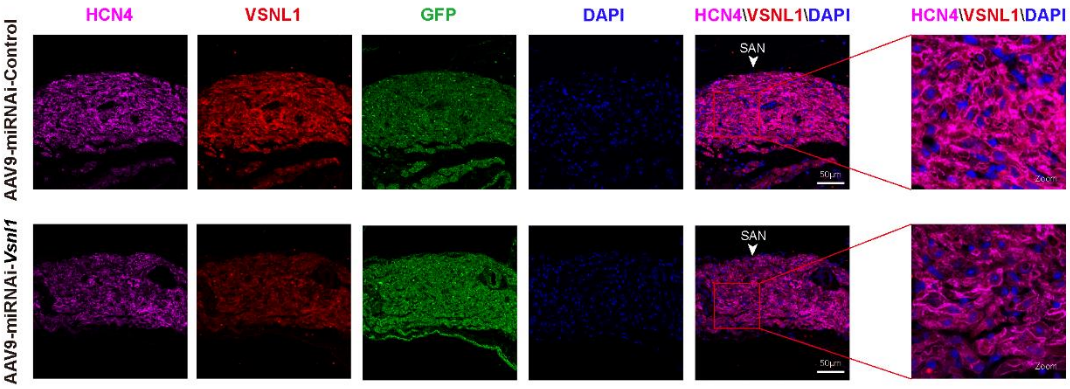

Supplementary Fig. 9. The knockdown of VSNL1 was showed in AAV9-miRNAi-*Vsnl1* mouse sinoatrial node (SAN) section. Immunostaining shows the co-staining of VSNL1 and HCN4 antibody in AAV9-miRNAi-Control and AAV9-miRNAi-*Vsnl1* mouse SAN sections (Scale bar = 50 μm). Arrowed heads point to SAN. The zoom images showed high magnification of the regions in red square. Representative images are shown from n = 4 biologically independent samples.

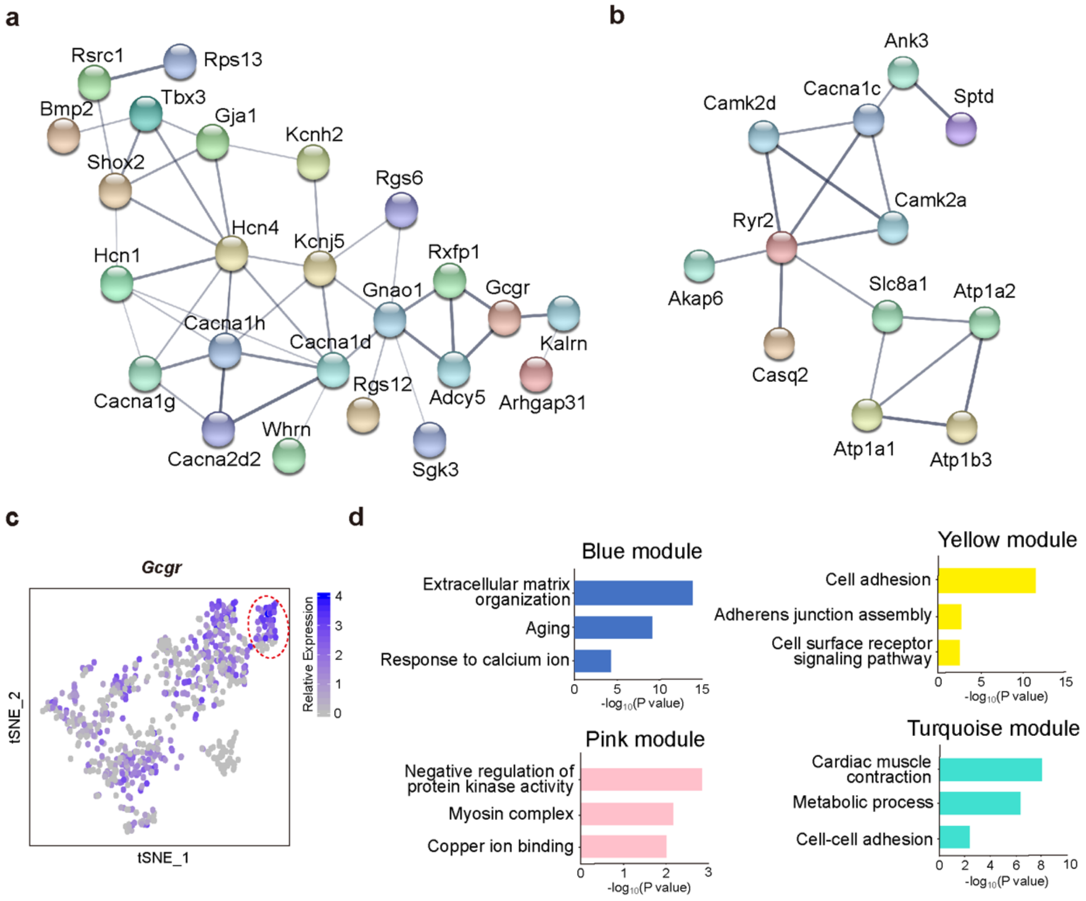

Supplementary Fig. 10. Functional analysis of mouse weighted gene co-expression network analysis (WGCNA) module genes. **a**, **b** Predicted and integrated protein-protein interaction network of red (**a**) and brown (**b**) module genes created by STRING, respectably. **c** Feature plot of *Gcgr* gene shows it was also mainly enriched in core cell clester (Cluster 4, indicated by the red circle), while it was involved in the gene co-expression network of red module and interacted with *Rxfp1* and *Adcy5* (**a**). **d** Gene Ontology (GO) analysis of blue, yellow, pink and turquoise modules shows these modules were also related to ion channels regulation but had certain difference.

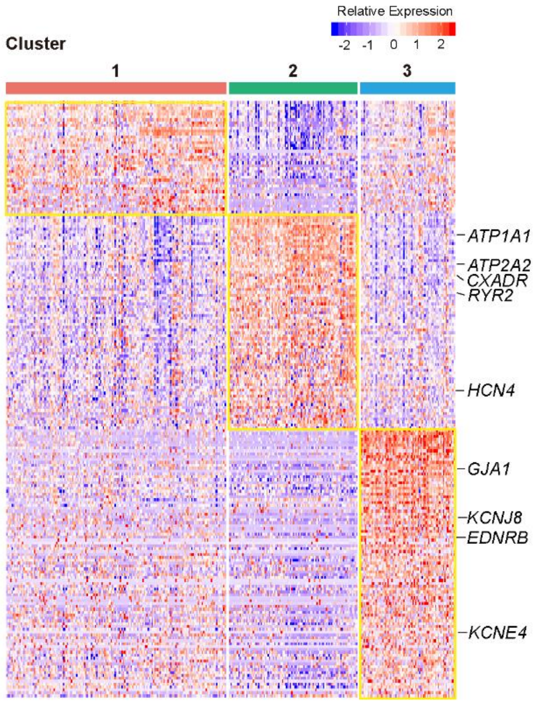

Supplementary Fig. 11. Heat map shows all differentially expressed genes (DEGs) of each cluster in rabbit sinoatrial node (SAN) cells.

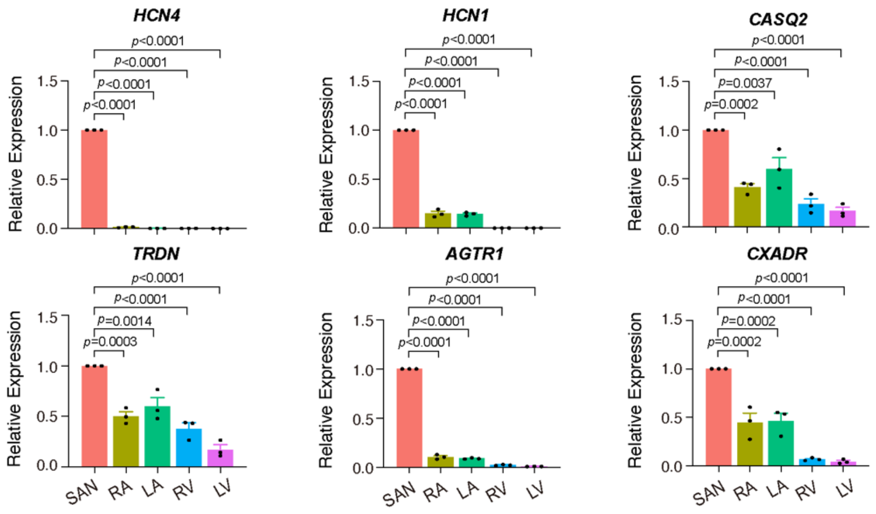

Supplementary Fig. 12. qPCR shows the expression of the core cell cluster genes in rabbit sinoatrial node (SAN), atrial and ventricular tissue, respectively (n = 4 biologically independent animals). RA, right atrium; LA, left atrium; RV, right ventricle, LV, left ventricle. Dunnett's multiple comparisons test. Data are represented as mean ± sem, adjusted p value was labeled on the top.

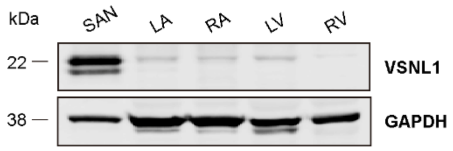

Supplementary Fig. 13. The expression of VSNL1 in rabbit sinoatrial node (SAN), atrial and ventricular tissues. Western Blot analysis shows the specific expression of VSNL1 in rabbit SAN. LA, left atrium; RA, right atrium; LV, left ventricle; RV, right ventricle.

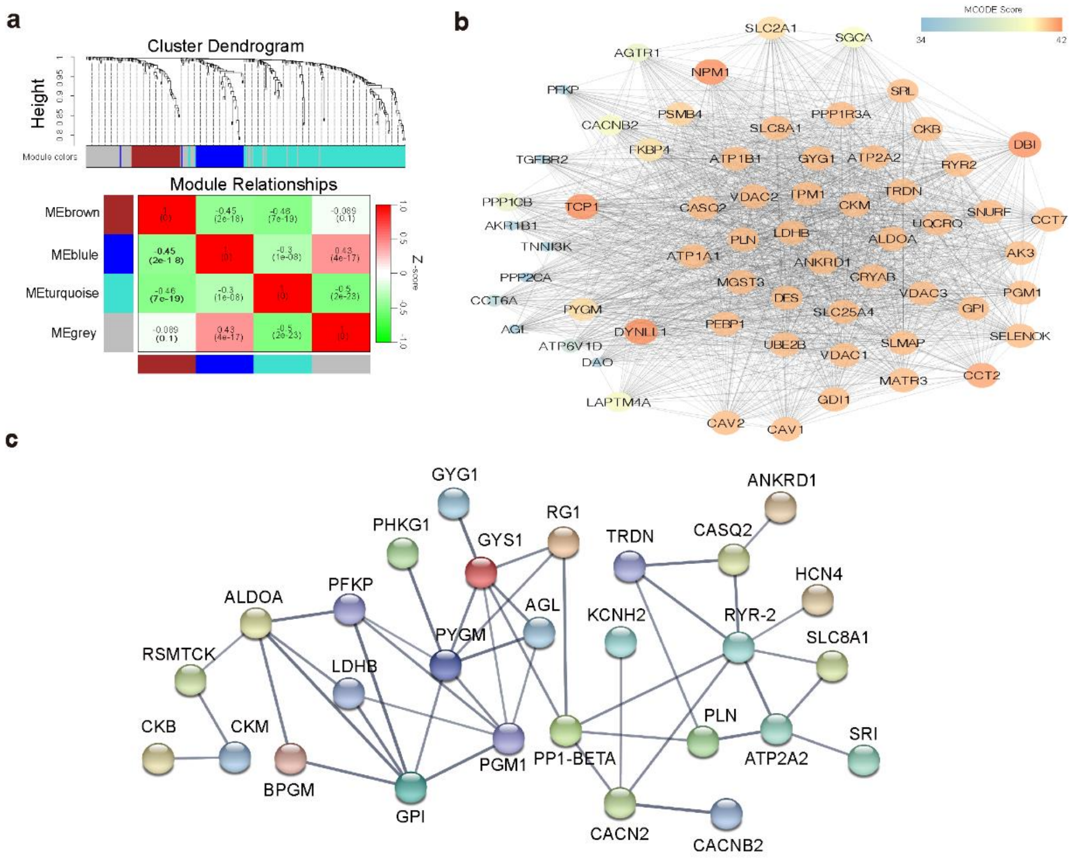

Supplementary Fig. 14. Weighted gene co-expression network analysis (WGCNA) analysis of rabbit sinoatrial node (SAN) cells. **a** The hierarchical cluster dendrogram identifies four gene co-expression modules by WGCNA. Heat map shows the modules were relative independence. **b** Co-expression network of hub genes in turquoise module created by Cytoscape mainly contained transporters and Ca^{2+} regulators. The high MCODE score of genes was mapped to the bright color of nodes. **c** Predict and integrated protein-protein interaction in turquoise module by STRING.

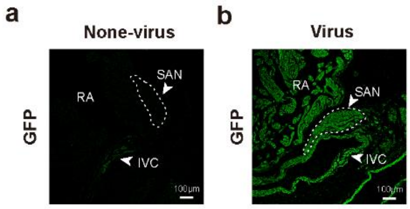

Supplementary Fig. 15. Fluorescence microscopy confirmed the AAV2/9-CMV-miRNAi-eGFP infection in mouse sinoatrial node (SAN). **a** The heart slice of control mouse not infected with virus. **b** the heart slice of mouse infected with virus. Dotted lines marked the SAN region; arrowed heads point to SAN or inferior vena cava (IVC). Representative images are shown from n = 3 biologically independent samples. Scale bar = 100 μm. RA, right atrium.

Supplementary Table 1. The siRNA sequences used for the gene knockdown experiments.

Gene	siRNA sequence
VSNL1-1	GCAAGAUGGAUAAGAACAATT
	UUGUUCUUAUCCAUCUUGCTT
VSNL1-2	GCAGUGCGACAUUCAGAAATT
	UUUCUGAAUGUCGCACUGCTT
RYR3	GGUCGUGGCUGAGAACUAUTT
	AUAGUUCUCAGCCACGACCTT
APOLD1	UGCGAGAGAUCCUGAGCUGTT
	CAGCUCAGGAUCUCUCGCATT
COL1A1	GCAAGACAGUGAUUGAAUATT
	UAUUCAAUCACUGUCUUGCTT
