# Supplementary material for: Cellular and molecular landscape of mammalian sinoatrial node revealed by single-cell RNA sequencing
Source: Nat Commun. 2021 Jan 12;12:287. doi: 10.1038/s41467-020-20448-x (PMC7804277; doi:10.1038/s41467-020-20448-x)
Supplement: Supplementary file 2 — Description of Additional Supplementary Files [file 41467_2020_20448_MOESM2_ESM.pdf]

Description of Additional Supplementary Files

File Name: Supplementary Data 1
Description: Heart related genes enriched in mouse SAN and RA reported previously. These differentially expressed genes (DEGs) were identified through comparing their expression in SAN and RA using RNA-seq data. SAN: sinoatrial node, RA: right atrium.

File Name: Supplementary Data 2
Description: Differentially expressed genes (DEGs) of 5 mouse SAN and AV cell clusters. These genes were identified through comparing their expression in a cluster with all other clusters using "FindMarkers" function in Seurat R package. The adjusted p-value based on bonferroni correction. SAN: sinoatrial node, AV: atrial and ventricular cardiomyocytes.

File Name: Supplementary Data 3
Description: Mouse genes extracted from the red and brown gene modules of WGCNA. Enriched GO terms of these genes were shown in Figure 5b. The hub genes which has the maximum connections with other genes in these two modules were identified and the gene expression networks were shown in Figure 5c, 5d. WGCNA: weighted gene co-expression network analysis.

File Name: Supplementary Data 4
Description: Differentially expressed genes (DEGs) of 3 rabbit SAN clusters. These genes were identified through comparing their expression in a cluster with all other clusters using "FindMarkers" function in Seurat R package. The adjusted p-value based on bonferroni correction.

File Name: Supplementary Data 5
Description: Rabbit genes extracted from the turquoise gene module of WGCNA. This module contains key genes encoding ion channels, transporters, Ca^{2+} regulators and others. WGCNA: weighted gene co-expression network analysis.

File Name: Supplementary Data 6
Description: Differentially expressed genes (DEGs) of 3 monkey SAN clusters. These genes were identified through comparing their expression in a cluster with all other clusters using "FindMarkers" function in Seurat R package. The adjusted p-value based on bonferroni correction.

File Name: Supplementary Data 7
Description: The primer sequences used for the qPCR detections in this study. Primers used for different species were shown separately.
