# Supplementary material for: Cellular and molecular landscape of mammalian sinoatrial node revealed by single-cell RNA sequencing
Source: Nat Commun. 2021 Jan 12;12:287. doi: 10.1038/s41467-020-20448-x (PMC7804277; doi:10.1038/s41467-020-20448-x)
Supplement: Supplementary file 10 — Reporting Summary [file 41467_2020_20448_MOESM10_ESM.pdf]

nature research

Corresponding author(s): Yi-Han Chen
Last updated by author(s): Nov 10, 2020

Reporting Summary

Nature Research wishes to improve the reproducibility of the work that we publish. This form provides structure for consistency and transparency in reporting. For further information on Nature Research policies, see Authors & Referees and the Editorial Policy Checklist.

Statistics

For all statistical analyses, confirm that the following items are present in the figure legend, table legend, main text, or Methods section.

n/a	Confirmed	
☐	☒	The exact sample size (n) for each experimental group/condition, given as a discrete number and unit of measurement
☐	☒	A statement on whether measurements were taken from distinct samples or whether the same sample was measured repeatedly
☐	☒	The statistical test(s) used AND whether they are one- or two-sided<br>*Only common tests should be described solely by name; describe more complex techniques in the Methods section.*
☒	☐	A description of all covariates tested
☒	☐	A description of any assumptions or corrections, such as tests of normality and adjustment for multiple comparisons
☐	☒	A full description of the statistical parameters including central tendency (e.g. means) or other basic estimates (e.g. regression coefficient) AND variation (e.g. standard deviation) or associated estimates of uncertainty (e.g. confidence intervals)
☐	☒	For null hypothesis testing, the test statistic (e.g. F, t, r) with confidence intervals, effect sizes, degrees of freedom and P value noted<br>*Give P values as exact values whenever suitable.*
☒	☐	For Bayesian analysis, information on the choice of priors and Markov chain Monte Carlo settings
☒	☐	For hierarchical and complex designs, identification of the appropriate level for tests and full reporting of outcomes
☒	☐	Estimates of effect sizes (e.g. Cohen's d, Pearson's r), indicating how they were calculated

Our web collection on statistics for biologists contains articles on many of the points above.

Software and code

Policy information about availability of computer code

Data collection	scRNA-seq: Illumina HiSeq X10 system.<br>qPCR: QuantStudioTMReal-Time PCR Software v1.2.<br>The telemetry ECG recording: DSI PhysioTel ETA-F10, ETA-F20, DSI Ponemah V6.30.<br>Microelectrode array (MEA) recording: Maestro Edge multiwell microelectrode array (MEA), AxIS Navigator 2.0.4.<br>Confocal microscopy: Leica Application Suite X, 3.5.19976.<br>Microscopy: Leica M205FA, Leica Application Suite v3.8.
Data analysis	Sequencing reads were separately aligned Hisat2 (version 2.0.5). The gene annotation file for mouse was downloaded from the database GENCODE (release 20), the RefSeq Genes tracks for cynomolgus monkey and rabbit genome from UCSC were exported to be their gene annotation files using the table tool. Finally the data were analysised by Seurat (version 2.3), DAVID (version 6.8), WGCNA (version 1.64), Cytoscape (version 3.6.1), MCODE (version 1.5.1) and STRING (version 11.0). qPCR, ECG and hiPSC-CMs beating rate were analysis using GraphPadPrism 8.

For manuscripts utilizing custom algorithms or software that are central to the research but not yet described in published literature, software must be made available to editors/reviewers. We strongly encourage code deposition in a community repository (e.g. GitHub). See the Nature Research guidelines for submitting code & software for further information.

Data

Policy information about availability of data

All manuscripts must include a data availability statement. This statement should provide the following information, where applicable:

- Accession codes, unique identifiers, or web links for publicly available datasets
- A list of figures that have associated raw data
- A description of any restrictions on data availability

The single-cell RNA-seq data have been deposited in the NCBI Sequence Read Archive (accession number of BioProject is PRJNA531288 [https://www.ncbi.nlm.nih.gov/bioproject/?term=PRJNA531288]). The codes used in this study are available in Github [https://github.com/xue-lab/scRNA-seq-of-sinoatrial-

node]. Figures associated with the single-cell RNA-seq data are: Figure 1, 5-8, Supplemental Figure 2, 3, 10, 11, 14. The data supporting the findings of this study are available within the article and Supplementary Information files. All remaining data will be available from the corresponding author upon reasonable request.

Field-specific reporting

Please select the one below that is the best fit for your research. If you are not sure, read the appropriate sections before making your selection.

☒ Life sciences ☐ Behavioural & social sciences ☐ Ecological, evolutionary & environmental sciences

For a reference copy of the document with all sections, see nature.com/documents/nr-reporting-summary-flat.pdf

Life sciences study design

All studies must disclose on these points even when the disclosure is negative.

Sample size	No statistical methods were performed to determine sample size. We obtained hundreds of sinoatrial node single cells from different species of mammals using manual cell picking for the subsequent clustering and WGCNA analysis. 771 cells including 718 SAN cells from 21 adult mice and 53 atrial and ventricular cardiomyocytes from 4 adult mice, 343 SAN cells from 6 adult rabbits, 296 SAN cells from 12 adult cynomolgus monkeys.<br>Sample size in other experiments was determined based on standards in the field and experiments to obtain statistical significance and reproducibility. At least triplicates were used to meet the minimal requirements for statistical analysis and the detailed sample size was demonstrated in the figure legends.
Data exclusions	Animals with unsufficient quality of the ECG in telemetry recordings were excluded from the study. The exclusion criteria were pre-established.
Replication	Smart-seq2 single-cell RNA-seq experiments were performed once.<br>qPCR experiments and Immunohistology analysis was performed at least three times independently.<br>Western blot analysis from SAN, atrium and ventricle tissues of rabbit was performed three times.<br>Beating rate of hiPSC-CMs recording experiments were performed at least three times.<br>Mouse ECG recording experiments were performed once with all biologically independent animals.<br>All attempts at replication were successful.
Randomization	All animals and cells were randomly assigned to the experimental group.
Blinding	The telemetry ECG recording was performed in a blind way, as the injection of virus, the measurement and data analysis were performed by different researchers.<br>The qPCR, histological experiments, western blots and the recording of hiPSC-CMs beating rate were performed blinded, as the samples/cells preparation by one researcher while subsequent measurement, data acquired and analysis were done by other researchers.

Reporting for specific materials, systems and methods

We require information from authors about some types of materials, experimental systems and methods used in many studies. Here, indicate whether each material, system or method listed is relevant to your study. If you are not sure if a list item applies to your research, read the appropriate section before selecting a response.

Materials & experimental systems

n/a	Involved in the study	
☐	☒	Antibodies
☐	☒	Eukaryotic cell lines
☒	☐	Palaeontology
☐	☒	Animals and other organisms
☒	☐	Human research participants
☒	☐	Clinical data

Methods

n/a	Involved in the study	
☒	☐	ChIP-seq
☒	☐	Flow cytometry
☒	☐	MRI-based neuroimaging

Antibodies

Antibodies used	Primary antibodies used in Immunofluorescence experiments:<br>1. HCN4: Sigma, cat.#SAB5200035, Monoclonal S114-10, 1:50 dilution<br>2. Connexin 43: CST,cat.#3512, Polyclonal, 1:50 dilution<br>3. VSNL1: Gene Tex, cat.#GTX115039, Polyclonal, 1:50 dilution<br>4. Collagen I: Abcam, ab21286, Polyclonal, 1:50 dilution<br>5. DLGAP1: Affbiotech, cat.#AF0308, Polyclonal, 1:50 dilution<br>6. UNC80: Bioss, cat.#bs-12121R, Polyclonal, 1:50 dilution<br>7. APOLD1: Novus Biologicals, cat.#NBP2-58460, Polyclonal, 1:50 dilution<br>8. RYR3: Novus Biologicals, cat.#NBP2-76962, Polyclonal, 1:50 dilution

	9. Connexin 40: Invitrogen, cat.#37-8900, Monoclonal, 1:50 dilution<br>10. cTNT: Abcam, cat.#ab8295, Monoclonal, 1:50 dilution<br><br>Primary antibodies used in Western Blots experiment:<br>1. VSNL1: Gene Tex, cat.#GTX115039, Polyclonal, 1:1000 dilution<br>2. GAPDH: Proteintech, cat.#60004-1-Ig, Monoclonal, 1:8000 dilution<br><br>Secondary antibodies included:<br>1. Goat anti-Mouse IgG Alexa Fluor® 488: Abcam, ab150113, 1:200 dilution<br>2. Goat anti-Rabbit IgG Alexa Fluor® 555: Abcam, ab150078, 1:200 dilution<br>3. Goat anti-Mouse IgG Alexa Fluor® 633: Sigma, SAB4600139, 1:200 dilution<br>4. Goat anti-Mouse IgG (H+L) Highly Cross-Adsorbed Secondary Antibody, Alexa Fluor Plus 800: Invitrogen, A32730, 1:10000 dilution<br>5. Goat anti-Rabbit IgG (H+L) Highly Cross-Adsorbed Secondary Antibody, Alexa Fluor Plus 800：Invitrogen, A32735, 1:10000 dilution
Validation	All following primary antibodies used in this study were validated and were cited in the litterature.<br>1. HCN4: https://www.sigmaaldrich.com/catalog/product/sigma/sab5200035?lang=zh&region=CN<br>Ref: Hughes et al. HCN4 subunit expression in fast-spiking interneurons of the rat spinal cord and hippocampus. Neuroscience. 237: 7-18 (2013).<br>2. Connexin 43: https://www.cellsignal.cn/products/primary-antibodies/connexin-43antibody/3512?N=4294956287&Ntt=3512&fromPage=plp<br>Ref: Zhong et al. Single cell transcriptomics identifies a unique adipose lineage cell population that regulates bone marrow environment. Elife. 9: e54695 (2020).<br>3. VSNL1: https://www.genetex.cn/Product/Detail/Visinin-like-1-antibody/GTX115039<br>Ref: Wu et al. The prevalence of CTNNB1 mutations in primary aldosteronism and consequences for clinical outcomes. Sci Rep. 7:39121 (2017).<br>4. Collagen I: https://www.abcam.cn/collagen-i-antibody-ab21286.html<br>Ref: Deng et al. Activation of hedgehog signaling in mesenchymal stem cells induces cartilage and bone tumor formation via Wnt/ß-Catenin. Elife. 8: e50208 (2019)<br>5. DLGAP1: http://www.affbiotech.com/goods-213-AF0308-DLGAP1+Antibody.html<br>6. UNC80: https://www.biossusa.com/products/bs-12121r<br>7. APOLD1: https://www.novusbio.com/products/apold1-antibody_nbp2-58460<br>8. RYR3: https://www.novusbio.com/products/ryr3-antibody_nbp2-76962<br>9. Connexin 40: https://www.thermofisher.com/cn/zh/antibody/product/Connexin-40-Antibody-clone-2F9A11-Monoclonal/37-8900<br>Ref: Boittin et al. Connexins and M3 muscarinic receptors contribute to heterogeneous Ca(2+) signaling in mouse aortic endothelium. Cell Physiol Biochem. 31(1):166-78 (2013).<br>10. cTNT: https://www.abcam.cn/cardiac-troponin-t-antibody-1c11-ab8295.html<br>Ref: Wu et al. LRP6 downregulation promotes cardiomyocyte proliferation and heart regeneration. Cell Res. (2020)<br>11. GAPDH: http://www.ptgcn.com/products/GAPDH-Antibody-60004-1-Ig.htm<br>Ref: Liu et al. Sox17 is required for endothelial regeneration following inflammation-induced vascular injury. Nat Commun. 10 (1): 2126 (2019)

Eukaryotic cell lines

Policy information about cell lines

Cell line source(s)	hiPSC-derived CMs were purchased from Help Stem Cell Innovations (Nanjing, China).<br>The HEK293 cell lines were from Key Laboratory of Arrhythmias of the Ministry of Education of China, Tongji University School of Medicine, Shanghai, China.
Authentication	hiPSC-derived CMs were authenticated prior to the experiments with flow cytometry, immunofluorescence staining and patch clamp.
Mycoplasma contamination	We have confirmed that the cells were tested negative for mycoplasma contamination.
Commonly misidentified lines<br>(See ICLAC register)	We did not use a commonly misidentified cell line in this study.

Animals and other organisms

Policy information about studies involving animals; ARRIVE guidelines recommended for reporting animal research

Laboratory animals	Male C57BL/6J mice (8-10 weeks, 6 and 12months), Male New Zealand White rabbits (6 and 18 months) and Male cynomolgus monkeys (8-15 years)
Wild animals	Not involved.
Field-collected samples	Not involved.
Ethics oversight	All of the animal experiments were approved by the Animal Care and Use Committee of Tongji University School of Medicine

Ethics oversight	(mouse and rabbit experiments), or Institutional Animal Care and Use Committee of Suzhou Xishan Zhongke Drugs Research and Development Centre (monkey experiments).

Note that full information on the approval of the study protocol must also be provided in the manuscript.
